# Characterization and Proteomic Profiling of Hepatocyte-like Cells Derived from Human Wharton’s Jelly Mesenchymal Stromal Cells: De Novo Expression of Liver-Specific Enzymes

**DOI:** 10.3390/biology14020124

**Published:** 2025-01-24

**Authors:** Melania Lo Iacono, Simona Corrao, Giusi Alberti, Giandomenico Amico, Francesca Timoneri, Eleonora Russo, Annamaria Cucina, Sergio Indelicato, Francesca Rappa, Tiziana Corsello, Salvatore Saieva, Antonino Di Stefano, Francesca Di Gaudio, Pier Giulio Conaldi, Giampiero La Rocca

**Affiliations:** 1 Department of Biomedicine, Neurosciences and Advanced Diagnostics (BiND), University of Palermo, 90127 Palermo, Italy; giusi.alberti@unipa.it (G.A.); francesca.rappa@unipa.it (F.R.); 2 Department of Biological, Chemical and Pharmaceutical Sciences and Technologies (STEBICEF), University of Palermo, 90128 Palermo, Italy; simona.corrao01@unipa.it; 3 Research Department, IRCCS ISMETT (Istituto Mediterraneo per i Trapianti e Terapie ad Alta Specializzazione), 90127 Palermo, Italy; gamico@fondazionerimed.com (G.A.); ftimoneri@fondazionerimed.com (F.T.); pgconaldi@ismett.edu (P.G.C.); 4 Unit of Regenerative Medicine and Immunotherapy, Ri.MED Foundation, 90133 Palermo, Italy; 5 Departmental Faculty of Medicine, Saint Camillus International University of Health Sciences, 00131 Rome, Italy; eleonora.russo@unicamillus.org; 6 Department of Health Promotion, Mother and Child Care, Internal Medicine and Medical Specialties (PROMISE) University of Palermo, 90127 Palermo, Italy; annamaria.cucina@unipa.it (A.C.); nondelicato@gmail.com (S.I.); francesca.digaudio@unipa.it (F.D.G.); 7 The Institute of Translational Pharmacology, National Research Council of Italy (CNR), 90146 Palermo, Italy; 8 Department of Pediatrics, Division of Clinical and Experimental Immunology and Infectious Diseases (CEIID), University of Texas Medical Branch, Galveston, TX 77550, USA; ticorsel@utmb.edu; 9 Department of Neurology, University of Texas Health Science Center at Houston, Houston, TX 77030, USA; salvatore.saieva@uth.tmc.edu; 10 Laboratory of Cardio-Respiratory Apparatus Cytoimmunopathology, “S. Maugeri” Foundation, IRCCS, Medical Center of Veruno, 281010 Novara, Italy; antonino.distefano@icsmaugeri.it

**Keywords:** Wharton’s jelly mesenchymal stromal cells, umbilical cord, perinatal stem cells, hepatocyte differentiation, liver diseases, hepatic failure, immune modulation, cell therapy, hypoimmunogenicity

## Abstract

End-stage liver disease (ESLD), affecting millions worldwide, represents a challenging issue for clinical research and global public health. Liver transplantation is the gold standard therapeutic approach but shows some drawbacks. Hepatocyte transplantation could be a reliable alternative for patient treatment. Mesenchymal stromal cells derived from Wharton’s jelly of the umbilical cord (WJ-MSCs) can differentiate into hepatocyte-like cells (HLCs) and show immunomodulatory functions. Due to the increasing demand for fully characterized cell therapy vehicles warranting both the safety and efficacy of treatments, in this work, we extensively characterized WJ-MSCs before and after the application of a hepatocyte-directed differentiation protocol. HLCs exhibited a morphology resembling that of hepatocytes, expressed early and late hepatic markers (α-fetoprotein, albumin, CK18, HNF4-α), and acquired hepatic functions (glycogen synthesis, xenobiotics detoxification), as also revealed by the shotgun proteomics approach. HLCs maintained the same pattern of immunomodulatory molecule expression and mesenchymal markers, other than displaying specific enzymes, suggesting these cells as promising candidates for cellular therapy of ESLD. Our work shed new light on the basic biology of HLCs, suggesting new therapeutic approaches to treat ESLD.

## Simple Summary

Liver transplantation is the only therapeutic option for patients affected by end-stage liver disease (ESLD), but its feasibility is restricted by the shortage of donated organs. Stem cell therapy could represent a valid treatment option for these conditions. In particular, the mesenchymal stromal cells derived from the human umbilical cord matrix (WJ-MSCs), thanks to their specific features, can be considered good candidates for liver tissue repair and regeneration. We found that the WJ-MSCs differentiate in hepatocyte-like cells (HLCs) using a differentiation protocol that resembles the in vivo liver morphogenesis. We also showed that HLCs acquired mature hepatic functions and maintained their intrinsic hypoimmunogenicity, a key feature to avoid rejection in the host. Moreover, thanks to proteomic analysis, we highlighted several hepatic proteins in HLCs, demonstrating that these cells could be valid candidates for the treatment of ESLD.

## 1. Introduction

Liver diseases represent a major human health problem affecting millions of people worldwide. To date, liver transplantation represents the only clinically effective treatment for end-stage liver disease (ESLD) [1], but, unfortunately, there are several disadvantages associated with this procedure: rejection of the transplanted organ, lack of usable organs, and high costs [2]. For these reasons, cell therapy is a viable strategy for the treatment of ESLD [3]. The availability of primary hepatocytes and, more recently, hepatocyte-like cells (HLCs) derived from various stem cells opens new avenues for cure. The two main functional effects commonly associated with stem cell therapy, repopulation and the bystander effect, are both desirable for the treatment of ESLD. These may be characterized by a failure of the organ’s self-repair mechanisms that are dampened by the underlying disease or lead to local depletion of stem cells. The success of cell therapy relies particularly on its ability to take over the functions of hepatocytes, as well as modulation of the local microenvironment, attenuation of inflammatory/remodeling processes, and immune cell activity (in the recent literature, these activities are generally referred to as “immunomodulatory activities”) [4].

A major challenge for cell therapies, especially in organ transplantation or tissue regeneration, is the ability of the transplanted cells to successfully integrate into the host tissue and survive there in the long term. In many cases, rejection by the immune system or implantation failure can undermine the therapeutic potential of the transplanted cells. MSCs have immunomodulatory properties that may favor the creation of a more tolerant immune environment. MSCs can suppress the activity of immune cells and reduce the production of inflammatory cytokines. For this reason, transplantation of MSCs may, on the one hand, help to improve the long-term survival of the transplanted cells, making it less likely that the recipient’s immune system will attack them. On the other hand, this means that patients may not need to rely on strong immunosuppressive drugs, which have significant long-term side effects.

Therefore, MSCs offer a double benefit in cell therapy: they promote effective tissue repair and regeneration and at the same time can reduce harmful dependence on immunosuppressive drugs, leading to better outcomes and fewer complications for patients [3,4].

Despite the success of primary hepatocyte transplantation, which is widely used for liver failure and liver metabolic disorders, there is still a discrepancy between the clinical need for hepatocytes and the scarcity of donor livers. Stem cells derived from various adult or perinatal tissues can differentiate into HLCs, acquiring mature hepatocyte functions, and can reverse the disease after transplantation [5,6,7].

In this context, MSCs are considered a very effective combination in the cell therapy of liver diseases thanks to their multipotency and immunomodulatory capacity, as reported in the literature [8,9]. MSCs can differentiate into hepatocytes under appropriately modulated conditions, in the presence of certain growth factors or microenvironments. Although their ability to directly regenerate the damaged liver is still the subject of active research, MSCs have been shown to enhance liver regeneration in various experimental models of liver disease. On the one hand, MSCs can contribute to the restoration of damaged liver tissue by stimulating the proliferation of native liver cells and promoting tissue repair through the secretion of growth factors such as VEGF (vascular endothelial growth factor) and HGF (hepatocyte growth factor). On the other hand, MSCs can differentiate into hepatocytes and contribute, albeit partially, to the restoration of selected liver functions [9].

The ability of MSCs to modulate the immune response is one of the main reasons rendering these cells promising for the treatment of chronic inflammatory liver diseases.

In conditions such as hepatitis or cirrhosis, chronic inflammation is a key factor leading to progressive liver damage. MSCs can inhibit the activation of immune cells (such as T lymphocytes and macrophages) and modulate the production of inflammatory cytokines. This may help reduce liver inflammation, a crucial factor in preventing additional damage. MSCs can induce an immunosuppressive environment through the secretion of molecules such as PGE2 (prostaglandin E2), IDO (indolamine 2,3-dioxygenase), and TGF-β (transforming growth factor beta), as well as expressing key membrane-bound molecules. These features may have a positive effect in reducing the abnormal immune response that can progressively damage the liver [10,11].

Another advantage of MSCs is their ability to prevent rejection in the case of liver transplantation. MSCs can reduce the activity of T lymphocytes that are responsible for transplant rejection, making liver transplantation safer and more durable in patients with end-stage liver failure [12,13]. Albeit further research is needed to fully understand the mechanisms at play and optimize therapeutic protocols, the use of MSCs represents a promising frontier in regenerative medicine for the liver.

There are a plethora of biological sources for MSCs, either adult, fetal, or perinatal tissues (such as umbilical cord and amniotic membrane) [14,15,16,17].

The umbilical cord (UC), one of the main sources of MSCs, is known to be composed of a specialized connective tissue called Wharton’s jelly (WJ) that surrounds the vessels involved in fetal circulation. WJ consists mainly of an amorphous substance rich in glycosaminoglycans, collagen fibers, and various cell types. In particular, the spindle-shaped WJ-MSCs are considered a promising cell type for tissue repair and regeneration [18].

The isolation of WJ-MSCs is technically simple, non-invasive, and ethically acceptable [19]. In culture, WJ-MSCs show excellent proliferation and differentiation properties [20]. It has been demonstrated that WJ-MSCs can differentiate into mature cells of all three embryonic layers under certain culture conditions [21,22] and are, therefore, desirable candidates for tissue repair and regeneration [23]. In addition, WJ-MSCs have been shown to be able to support hematopoietic stem cell proliferation in vitro [24] and to survive in in vitro models of diseases characterized by hypoxic conditions [25].

Importantly, WJ-MSCs have key immunological properties due to their physiological localization in placental tissue. They express both canonical (HLA-A-B-C) and non-canonical (HLA-E, F, and G) MHC class I antigens [26,27,28] and do not feature MHC class II molecules (HLA-DR, DP, and DQ). In addition, WJ-MSCs do not express costimulatory antigens involved in the activation of T and B cell responses, such as CD40/CD40L, CD80, CD86, and B7-DC [18]. However, the expression of some of these antigens is restricted to MSCs stimulated with INF-α or other stimulators [29,30,31,32]. Most of these molecules are a common feature of MSCs from other tissues [33,34].

We have previously shown that WJ MSCs do not express costimulatory molecules after differentiation [26]. This is probably linked to the non-immunogenic nature of these cells. It has been reported that the expression of B7-H1, a negative regulatory molecule, can suppress T cell proliferation. WJ-MSCs also suppress the differentiation and maturation of monocytes into dendritic cells in a contact-dependent manner and promote the induction of differentiation/maturation of regulatory T cells (Tregs), confirming the effective immunomodulatory functions of these MSCs [35].

Several reports indicate that WJ-MSCs can be successfully differentiated into HLCs that exhibit features of mature hepatocytes [36]. Bharti et al. have shown that HLCs derived from WJ-MSCs exhibit high hepatogenic potential as well as the ability to produce urea, indicating ammonia competence [37]. However, a comprehensive characterization of the differentiated cells is lacking.

Based on all these considerations, in this study, we performed a specific differentiation protocol of WJ-MSCs into HLCs, followed by their characterization using different techniques and functional assays to highlight the similarity related to liver function, hepatic expression profile, and immunogenic properties. Our proteomic approach discovered several novel molecules specifically expressed after the differentiation of HLCs as well as quantitative differences between the different conditions described for the first time in these cells derived from the differentiation of stromal cells from perinatal tissues.

## 2. Materials and Methods

### 2.1. Cellular Isolation Protocol of WJ-MSCs

The isolation of WJ-MSCs was performed following our previously published protocol [26,27]. 10 human umbilical cords were obtained after the mother’s consent according to the tenets of the Declaration of Helsinki and local ethical regulations (IRRB/58/13, ISMETT Institutional Research Review Board). Briefly, after full-term birth through normal vaginal or cesarean delivery, umbilical cords were aseptically stored in a cold saline-buffered solution, and cellular isolation was started within six hours of birth. The cords were washed in warm HBSS (h9394, Sigma-Aldrich, Steinheim, Germany) with antibiotics/antimycotics (A5955, Sigma-Aldrich, Germany, concentration of use is 20 mL/L), and then they were cut into small pieces (about 1.5 cm length) and sectioned longitudinally to exhibit the WJ under the umbilical epithelium. Multiple incisions, without vessel removal, were made within the cord tissue with a sterile scalpel to increase the area open to contact with the plastic surface of 6-well plates. The cord pieces were cultured in the presence of a standard medium composed of DMEM low glucose medium (D5523, Sigma- Aldrich, Steinheim, Germany), supplemented with 10% fetal bovine serum (FBS gold, PAA), 1x NEAA (M7145, non-essential amino acids, Sigma-Aldrich, Germany), 1x antibiotics/antimycotics (A5955, Sigma-Aldrich, Germany), and 2 mM L-glutamine (G7513, Sigma-Aldrich, Germany). This isolation protocol is based on the natural migratory ability of mesenchymal cells and, therefore, the use of potentially harmful enzymatic activities can be skipped. Cord pieces were kept in culture for 15 days with medium change every second day. The slow degradation of the matrix allowed growth factors and signaling molecules to exit from the cord with a continuous positive stimulation of the cultured cells. After 15 days of culture, cord pieces were removed, and the adherent cells were routinely cultured until reaching 80% confluence.

### 2.2. Cell Culturing and Passaging

WJ-MSCs, at 80% confluence, were detached from flasks using TrypLe Select (12563, Tryple Select Enzyme, no phenol red, Gibco, Waltham, MA, USA) and were cultured for up to 15 passages. For immunocytochemical analysis and PAS staining, cells were plated in 8-well chamber slides (BD Bioscience, Leuven, Belgium). For flow cytometry analysis, cells were cultured in 25 cm^2^ tissue culture flasks (Corning, Berlin, Germany). For proteomic analysis, the cells were cultured in 75 cm^2^ tissue culture flasks to obtain an 80% confluence (2 × 10^6^ cells). WJ-MSCs were cultured in 96-well plates for CYP3A4 activity assay and in 12-well plates for a Glucose-6-Phosphatase assay.

### 2.3. Flow Cytometry (FC) Analysis

WJ-MSCs (1 × 10^5^) were stained in the presence of PBS (Sigma-Aldrich, Darmstadt, Germany) with 0.5 % BSA (A2153, Sigma-Aldrich, Hoeilaart, Belgium) with several antibodies, and their isotype match control was used as a negative control. The intracellular staining was performed using a BD Cytofix/Cytoperm™ solution (554714, BD Biosciences, Erembodegem, Belgium). The primary antibodies and isotype controls used for the FC analyses are summarized in Table S1. Forward and side scatter gates were set to include all viable cells. Routinely, debris and doublets were excluded from the cell population data by applying forward and side scatter selection. The co-expression of two markers was analyzed by the gating of the population for the first marker and the subsequent analysis of the percentages of the second marker. FC data were acquired with a BD FACS Aria II instrument. Data obtained by flow cytometer were acquired with FACS Diva 6.1.2, while the analysis was performed with the FlowJo^TM^ 10 version software.

### 2.4. Immunocytochemistry (ICC) Analysis

ICC analysis was performed as previously reported [27]. After culturing, cells grown in chamber slides were washed with PBS and fixed in methanol for 20 min at −20 °C. Air-dried slides were then stored at −20 °C until use. Cells were permeabilized with 0.1% Triton X-100 (1610407, Biorad, Hercules, CA, USA) in PBS (Sigma-Aldrich, Germany). After a subsequent rinse with PBS, slides were exposed to 0.3% hydrogen peroxide in PBS, blocked with 1% FBS in PBS, and incubated for 2 h with the primary antibody. The detection was performed using an avidin–biotin complex kit (LSAB2; Dako, Golstrup, Denmark). The 3-amino-9-ethylcarbazole (AEC chromogenic substrate solution; Dako, Golstrup, Denmark) was used as a developer. Nuclear counterstaining was obtained using hematoxylin (Dako, Golstrup, Denmark). Negative controls were used simultaneously by omitting the primary antibody step. The antibodies used in the present study, with indications of the working conditions, are listed in Table S2. CYP isoforms’ staining was analyzed and quantified for statistics using the open-source software Fiji (version 2.16), and the results were plotted and expressed as intensity based on the percentage (%) of the area showing a positive signal.

### 2.5. Immunohistochemistry (IHC) Analysis

Archival paraffin sections of the human liver with a thickness of 5 µm were used for IHC analysis. The staining was carried out using a streptavidin–peroxidase kit (LSAB2 system peroxidase; Dako, Golstrup, Denmark) following the manufacturer’s instructions. Briefly, after deparaffinization steps, the sections were exposed to 0.3% hydrogen peroxide solution for 10 min to inactivate endogenous peroxidases. A blocking solution (PBS with 3% BSA was used for 1 h at room temperature (RT). Specific primary antibodies used for this work (listed in Table S2) were applied for 1.5 h of incubation at RT. Sections without primary antibodies were used as negative controls and were run simultaneously. A 1X phosphate buffer saline (PBS) (Sigma-Aldrich, Germany) was used for the washing steps. Sections were then incubated with mouse/rabbit antibodies (universal secondary antibodies from the LSAB2 system kit) for 1 h. Subsequently, streptavidin–peroxidase was added, followed by 3-amino-9-ethylcarbazole (AEC) (Dako, Golstrup, Denmark) as a developer, and nuclei were counterstained using hematoxylin (Dako, Golstrup, Denmark).

### 2.6. Hepatocyte-Like Cells Differentiation Protocol

WJ-MSCs were used for differentiation experiments between the 5th and 7th passages. We developed a modified protocol based on existing reports about the usefulness of fibroblast growth factors (FGFs) and hepatocyte growth factor (HGF) as initial inducers of the hepatocyte-like phenotype [5,6,27]. For the first three weeks, the hepatic differentiation medium was composed of DMEM low glucose, 1% FBS (FBS Gold, PAA), 1x NEAA (M7145, Sigma-Aldrich, Germany), 1x antibiotics/antimycotics (A5955, Sigma-Aldrich, Germany), 2 mM L-glutamine (Sigma-Aldrich, Germany), 10 ng/mL of human FGF-4 (Miltenyi Biotech, Germany), 20 ng/mL of human HGF (Miltenyi Biotech, Germany), 1x Insulin-transferrin-selenite supplement (ITS, I3146, Sigma-Aldrich, Germany), and 0.1 µM dexamethasone (Sigma-Aldrich, Germany). Medium changes were performed each other day. After 3 weeks, the culture medium was further supplemented with 10 ng/mL of oncostatin M (OSM, Miltenyi Biotec, Bergisch Gladbach, Germany) for another week. For all the lines tested in differentiation experiments, the control WJ-MSCs were cultured in the standard medium as described above. Both the treated and control WJ-MSCs were analyzed at the end of the 3rd and 4th week of the hepatic differentiation process. Constant cell monitoring throughout the culture period was performed by phase-contrast microscopy. Treated cells were referred to as HLCs.

### 2.7. Glycogen Staining with Periodic Acid Schiff (PAS)

PAS staining was performed using a commercial kit (395B, Sigma-Aldrich, Germany) following the manufacturer’s instructions. Briefly, control WJ-MSCs and HLCs, grown into 8-well chamber slides, were fixed in formaldehyde/ethanol fixative solution for 1 min. After washing with tap water for 1 min, periodic acid was added for 15 min at room temperature. Following several washes with distilled water, Schiff’s reagent was added for 15 min at room temperature. Subsequently, another wash with running tap water was performed for 5 min to remove Schiff’s reagent, and the cells were stained with Gill’s hematoxylin for 90 s. Slides were then washed and air-dried, and pink-to-red glycogen deposits were observed at the light microscope.

### 2.8. Glucose-6-Phosphatase (G-6-Pase) Activity Assay

To detect G-6-Pase activity, we used a previously published protocol [38,39]. Briefly, both control and HLCs were incubated with a working solution composed of 2.4 mM lead nitrate and 2.08 mM glucose-6-phosphate in 0.1 M Tris-acetate buffer pre-warmed at 37 °C. After 20 min, the working solution was removed, and three washes were performed with distilled water. Then, 5% ammonium sulfide was added for 30 s to convert lead nitrate into lead sulfate, which is visible as brownish–black precipitates. Standard counterstaining was performed with hematoxylin prior to observing cells at the inverted microscope (DMIL, Leica, DMI 300 B, Wetzlar, Germany).

### 2.9. CYP450 3A4 Metabolic Activity Assay

The Kit P450-Glo™ CYP3A4 Assay (Luc-PFBE, Promega, Madison, WI, USA) was used to assess the induction and activity of CYP3A4 in HLCs following the manufacturer’s instructions. Briefly, cells were plated in 96-well plates and incubated for 48 h with 25 µM rifampicin (Sigma), which is a known inducer of the enzyme. In parallel experiments, cells were co-incubated with 10 µM ketoconazole (Sigma-Aldrich, Germany), which specifically inhibits CYP3A4 activity. Medium changes were performed daily. After washing with PBS, the cells were incubated with 50 µM of luciferin-PFBE, a luminogenic substrate, for 4 h in the dark. Subsequently, 50 µL of medium from each well was transferred to a 96-well opaque white luminometer plate, and 50 µL of luciferin detection reagent was added. After 20 min incubation at room temperature, samples were analyzed with a luminometer (Promega, Madison, WI, USA) using integration times of 0.5 s. To determine background luminescence, a luminogenic substrate was added to the complete medium without cells. Background subtraction was applied to all the samples, which led to an apparently “negative” luminescence value in NT-WJ-MSCs (reported in the relative figure).

### 2.10. Sample Preparation for Proteome Analyses

For the analysis of secreted proteins, the cells were detached with TrypLE Select (Gibco) at 37 °C for 3–5 min, washed with PBS, and lysed with NP-40-containing buffer (20 mM Tris/HCl pH 7.5, 1% Nonidet NP-40, 50 mM NaCl, 1x protease inhibitor). Desalting, buffer exchange, and protein concentration were carried out using 3K NMWL Amicon Ultra Centrifugal Filters 0.5 mL (Millipore, Darmstadt, Germany). Protein quantification was performed using DC Protein Assay (Biorad, Hercules, CA, USA).

### 2.11. In-Solution Digestion

The digestion step was performed by adding Trypsin/Lys-C Mix (Promega) to 50 μg of proteins at a 25:1 protein/protease ratio (*w*/*w*), following the manufacturer’s instructions. Peptides, obtained after the digestion, were concentrated and desalted with Pierce C18 Spin Columns (Thermo Scientific, Waltham, MA, USA). Then, the samples were lyophilized and suspended in an appropriate buffer (0.1% formic acid) for subsequent applications.

### 2.12. High-Performance Liquid Chromatography (HPLC) and Electrospray MS

Reversed-phase HPLC separation and online mass spectrometry detection (HPLC-MS/MS) were performed using an UltiMate 3000 System (Dionex, Sunnyvale, CA, USA) combined with Linear Trap Quadrupole (LTQ) Orbitrap XL™ Hybrid Ion Trap-Orbitrap Mass Spectrometer (Thermo Scientific, Milan, Italy) via an electrospray ionization (ESI) source. The chromatographic separation was carried out on a Hypersil Gold C18 column (15 cm × 2.1 mm, 1.9 μm). Sequential elution of peptides was accomplished using a flow rate of 50 μL min^−1^ and a linear gradient from 98% solution A (0.1% formic acid) to 98% solution B (80% acetonitrile, 0.08% formic acid) over 120 min. The gradient elution program was as follows: 2 min 98:2, 4 min 87:13; 95 min gradient from 87:13 to 50:50, 104 min 2:98, 109 min 2:98, 110 min 98:2, and 120 min 98:2 (A:B, *v*/*v*). The column temperature was set at 25 °C, and the sample tray temperature was maintained at 4 °C. For all experiments (n = 4 for each condition), a sample volume of 4 μL corresponding to 250 ng of peptides was loaded. For MS detection, the ESI source was operated in positive mode and run with Xcalibur version 2.0 (Thermo Fisher). High-purity nitrogen was used as the sheath and auxiliary gas, and high-purity helium was used as the collision gas. To limit the under-sampling effect, different orbital filling times (namely, 400 and 500 ms) were used.

### 2.13. Proteomic Data Analysis

The raw data were processed using Proteome Discoverer version 1.4 (Thermo Scien-tific, Waltham, MA, USA). MS/MS spectra were sequentially searched with Sequest HT and MS Amanda, both set against *Homo sapiens* and *Bos taurus* databases using the following parameters: full trypsin digestion with a maximum of two missed cleavages, fixed modification of carbamidomethylating of cysteine (+57.021 Da), variable modification of oxidation of methionine (+15.995 Da), and phosphorylation of tyrosine, serine, and threonine (+79.966 Da). Precursor mass tolerance was set at 10 ppm, and fragment ion tolerance was set at 0.8 Da. Peptide spectral matches were validated using the percolator software tool (http://percolator.ms/). Only proteins with ≥3 peptides matched were selected for subsequent data analyses.

### 2.14. In Silico Analysis

To confirm the identified proteins, in silico analyses were carried out. Notably, to avoid the detection of false positive molecules from FBS, we also compared the sequences of human candidate peptides against Bos taurus protein sequences using LALIGN (https://www.ebi.ac.uk/Tools/psa/lalign/) (accessed on 9 October 2024). The accession number of each identified protein was loaded into the gene ontology (GO) classification system (http://www.geneontology.org). The proteins were analyzed considering the different cellular components, molecular functions, and biological processes. However, the ‘molecular functions’ and ‘biological process’ were the only classifications used for generating pie charts and related statistics. Automatically, the PANTHER (Protein ANalysis THrough Evolutionary Relationships) GO classification system generated the pie charts with related statistics. General functions analysis was performed using the UniProt database (http://www.uniprot.org). The Human Protein Atlas (HPA) database (https://www.proteinatlas.org/humanproteome) (accessed on 9 October 2024) was used for mapping proteins in human organs, tissues, and cells based on omics technologies, including antibody-based imaging, mass spectrometry-based proteomics, transcriptomics, and systems biology.

### 2.15. Label-Free Quantification and Data Analysis

The raw data were analyzed by the software Maxquant version 2.4.2.0. The MS data were searched against the UniProt-reviewed database of *Homo sapiens*. A second UniProt-reviewed database of *Bos taurus* was added to identify potential contaminants from the cell medium. The common Repository of Adventitious Proteins (c-RAP) contaminant database was included in the database search. Proteins in common between *Homo sapiens* and *Bos taurus* were considered as contaminants and not further investigated. Proteins with at least three peptides were analyzed. A semi-quantitative analysis was performed by selecting the Fast label-free quantification (LFQ) option with a classic normalization. Only unique peptides were used for quantification. The LFQ of proteins required at least two ratio counts of unique peptides. “Advanced ratio estimation”, “stabilize large LFQ ratios”, “require MS/MS for comparisons”, and “advanced site intensities” were selected. The runs were automatically aligned. SM, Protein, and Site decoy fraction FDR were set at 0.05. The LFQ values were log2 transformed and statistically analyzed by using the Perseus software 2.0.10.0. A PCA test (*p*-value < 0.05) and two-sample t-student tests were performed to evaluate the differences between the 3rd and 4th week NT WJ-MSCs and the 3rd and 4th week HLCs. The results were plotted in a Volcano plot in which the x-axis presents the differences between (tr) and (nt) cells expressed as the log2(LFQ Intensity HLCs)/log2(LFQ Intensity NT WJ-MSCs), while the y-axis represents the significance derived from the −log10(*p*-values). With a FDR set at 0.05, only statistically significant upregulated and downregulated ones were considered. The proteins upregulated in HLCs were analyzed using the STRING database (https://string-db.org/, accessed on 9 October 2024) to define the protein-protein association networks.

### 2.16. Statistical Analyses

The obtained data were plotted using MS Excel software, and statistical analyses were performed on triplicate samples using GraphPad Prism 4 software (GraphPad Software, San Diego, CA, USA). The significance of differences between the experimental conditions was assessed by the Mann–Whitney test and one-way ANOVA analysis (Kruskal–Wallis test). Values were considered significant for *p* < 0.05.

## 3. Results

### 3.1. WJ-MSCs Show Mesenchymal Phenotype and Intrinsic Hepatogenic Potential

WJ-MSCs were cultured in standard medium on plastic surfaces and maintained a fibroblast-like morphology at low and high confluency. WJ-MSCs were routinely passaged and maintained in culture until the 15th passage.

**Figure 1:**
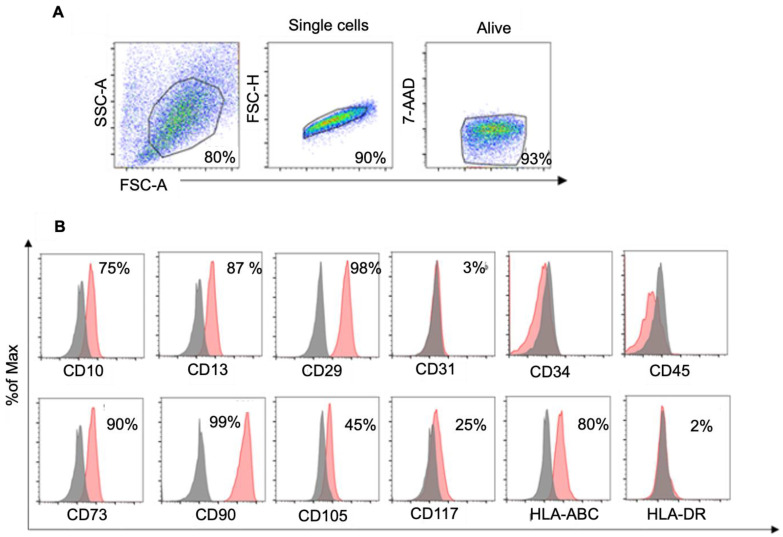
(**A**) Representative gate strategy for FC analysis; (**B**) representative FC analysis of several mesenchymal and hematopoietic/endothelial marker expressions in WJ-MSCS. The gray peaks represent the isotype control, while the red peaks reveal the percentage of specific marker expression.

We analyzed the expression of consensus mesenchymal stromal cell markers [40] as well as additional markers during cell culture. In naïve WJ-MSCs, we confirmed the expression of typical mesenchymal markers such as CD10, CD13, CD29, CD73, CD90, CD105, and CD117 by flow cytometry analysis. The presence of endothelial and hematopoietic markers (CD31 and CD34, CD45, respectively) was predominantly negative in these cells (Figure 1).

Confirming our previously published data [26], WJ-MSCs express, at the protein level, type I (major histocompatibility complex) MHC molecules, such as HLA-A, -B, and -C (class Ia HLA, human leukocyte antigen), but not HLA-DR, a type II MHC molecule (Figure 1). The standard multilineage differentiation potential of WJ-MSCs was demonstrated following our previously published protocols [26,27] (Figure S1).

**Figure 2:**
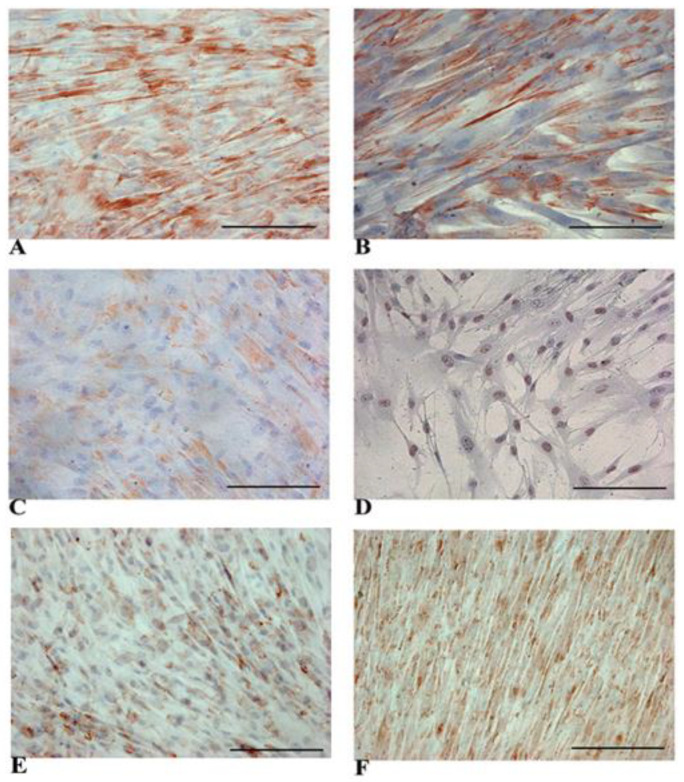
Representative panels of immunocytochemical detection of multiple hepatic tissue-specific markers in naïve WJ-MSCs. Micrographs show the expression of CK-8 (**A**), CK-18 (**B**), and CK-19 (**C**) at the cytoplasm level. HNF-4α showed a clear nuclear positivity (**D**). The expression of connexin 32 (**E**) and connexin 43 (**F**) showed a typical membrane staining. Magnification 20×. Scale bar: 100 µm.

As shown in Figure 2, naïve WJ-MSCs exhibited a clear cytoplasmic positivity for some liver-specific epithelial cytokeratins, namely CK-8 (A), CK-18 (B), and CK-19 (C). Figure 2D shows, for the first time, that WJ-MSCs expressed HNF-4α, a molecule directly involved in the induction of albumin and AFP expression [41].

It is known that up to seven forms of connexins, proteins involved in the formation of gap junctions at the cell surface, are expressed in different liver cells [42]. As shown in Figure 2E,F, we detected the expression of hepatocyte-specific connexins 32 and 43 in naïve WJ-MSCs with distinct membrane staining, suggesting that gap junctions normally found in the liver can be formed extensively between these cells [42,43]. The overall results emphasize the ability of WJ-MSCs to differentiate into hepatocyte-like cells (HLCs).

### 3.2. Evidence of Hepatic Phenotype Acquisition in WJ-MSCs After Hepatogenic Differentiation Protocol

**Figure 3:**
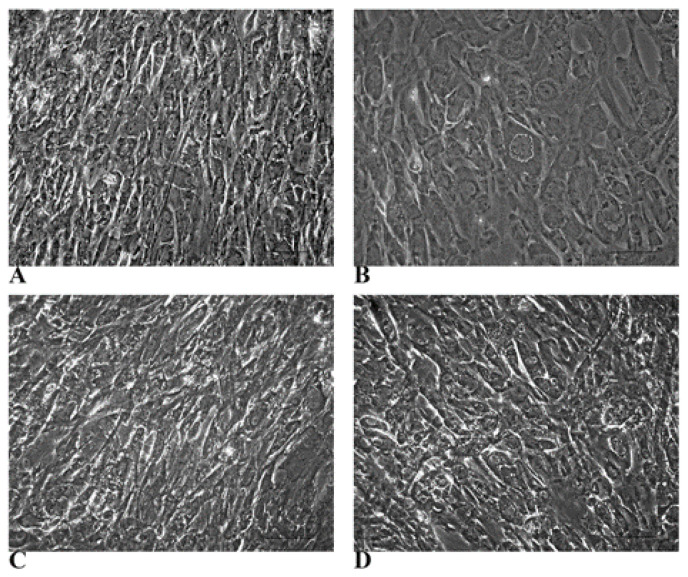
Microscopic demonstration of the morphological changes induced by differentiation and glycogen deposition. Phase-contrast micrograph panels show morphological changes between NT WJ-MSCs (**A**,**C**) and HLCs (**B**,**D**) at different time points of hepatic differentiation. (**A**,**B**): 3rd week; (**C**,**D**): 4th week. Magnification 20×. Scale bar: 100 µm.

Both HLCs and control WJ-MSCs (cultured in a standard medium; named from here as not treated NT WJ-MSCs) were analysed in the 3rd and 4th week of the differentiation process. Phase-contrast microscopy allowed us to determine a clear morphological switch in HLCs compared with NT WJ-MSCs (alongside the culturing process (Figure 3). HLCs showed a polygonal shape (resembling that of hepatocytes), while NT WJ-MSCs maintained their fibroblast-like morphology (Figure 3).

**Figure 4:**
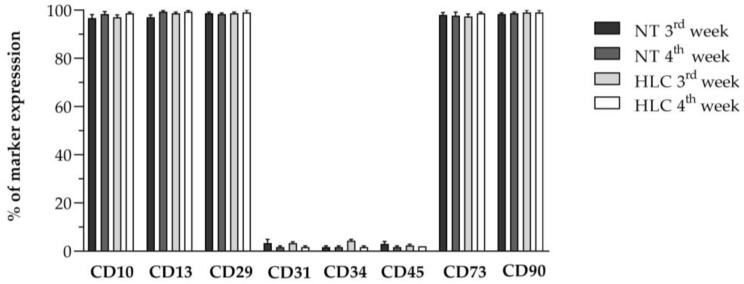
Flow cytometry analysis of the expression of canonical MSC markers and hematopoietic/endothelial markers after 3 and 4 weeks of hepatic differentiation in NT WJ-MSCs and HLCs. Data are represented as the percentage of marker expression. Data are the mean of three independent experiments. Comparisons among groups were made using the Kruskal–Wallis test. Statistical analysis among groups is non-significant.

Importantly, in Figure 4, we showed that HLCs still maintained the phenotype of the parental WJ-MSCs, in line with the observations originally made by Campard and colleagues [44].

**Figure 5:**
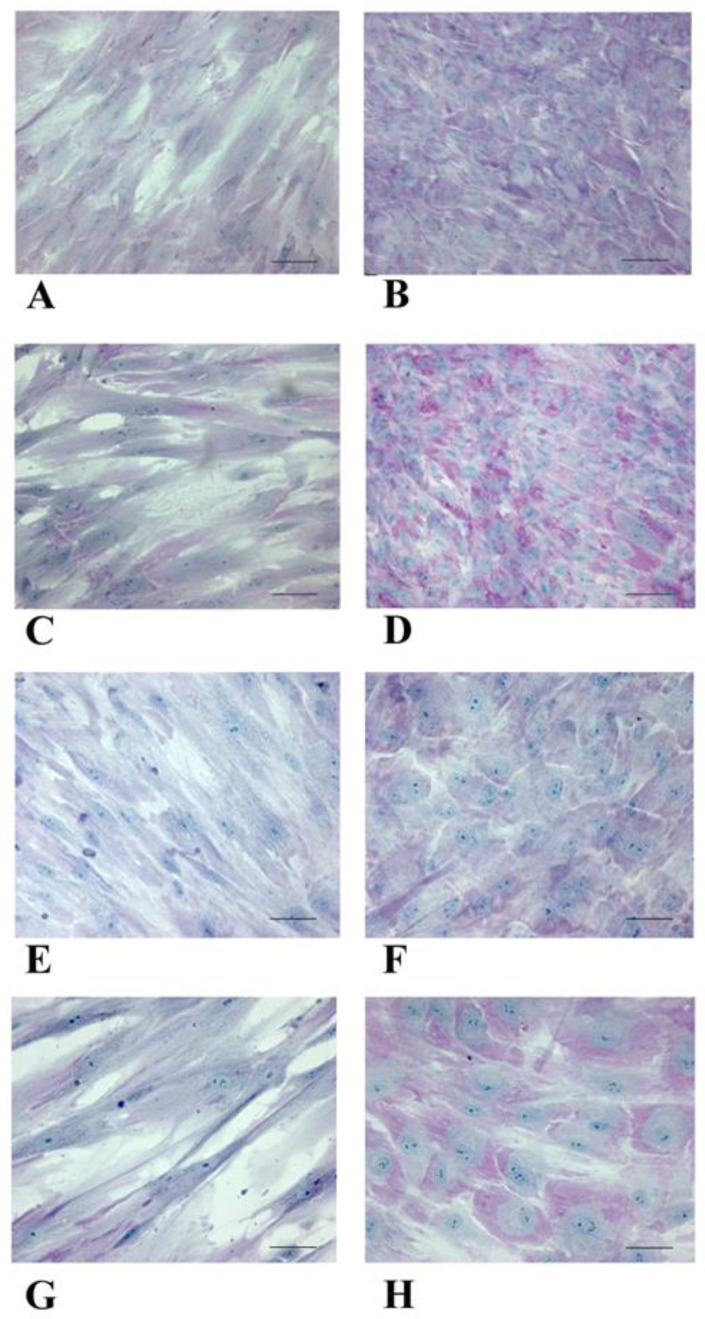
Micrographs panel shows PAS staining on naïve NT WJ-MSCs (**A**,**C**,**E**,**G**) and HLCs (**B**,**D**,**F**,**H**) at 3rd and 4th week after differentiation. (**A**,**B**,**E**,**F**): 3rd week; (**C**,**D**,**G**,**H**): 4th week. Magnification: (**A**–**D**): 20×; (**E**–**H**): 40×; Scale bar: (**A**–**D**): 100 µm; (**E**–**H**): 50 µm.

To further validate the hepatic-like phenotype acquisition by WJ-MSCs, we performed PAS staining. We observed that NT WJ-MSCs were negative to PAS staining, while HLCs showed an intense diffuse purple–magenta stain, strongly suggesting the presence of glycogen deposits (Figure 5).

**Figure 6:**
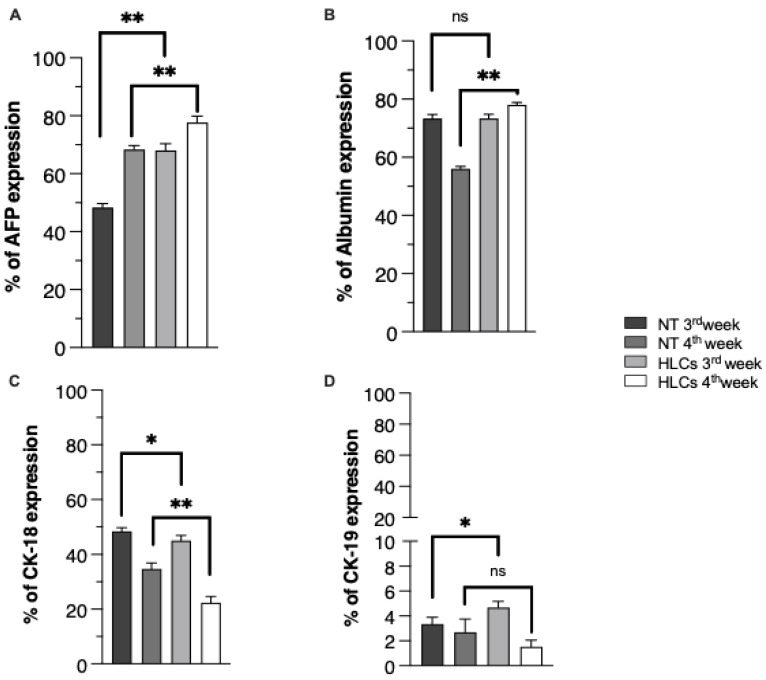
Hepatocyte differentiation effects on the expression of hepatocyte markers. Flow cytometry analysis was performed on NT WJ-MSCs and HLCs at 3rd and 4th week of hepatic differentiation protocol: AFP (**A**), Albumin (**B**), CK-18 (**C**), and CK-19 (**D**). Data are represented in percentage of marker expression. Data are mean of three independent experiments. Comparison among groups were made using Mann–Whitney test; ns, not significant; * *p* ≤ 0.05; **p ≤ 0.01.

While AFP expression may characterize early/fetal hepatocytes, albumin is expressed alongside maturation [39,41]. We showed that the expression levels of these proteins were maintained over the culturing and differentiation period (Figure 6A,B and Figure S2). Mature hepatocytes express CK-18, while they lack CK-19 [43]. Our results confirm this pattern in HLCs; as shown in Figure 6C and Figure S2, CK-18 was expressed in NT WJ-MSCs and HLCs at all time points, while CK-19 was expressed at lower levels with a tendency to decrease as a function of the differentiation in 4th week HLCs.

**Figure 7:**
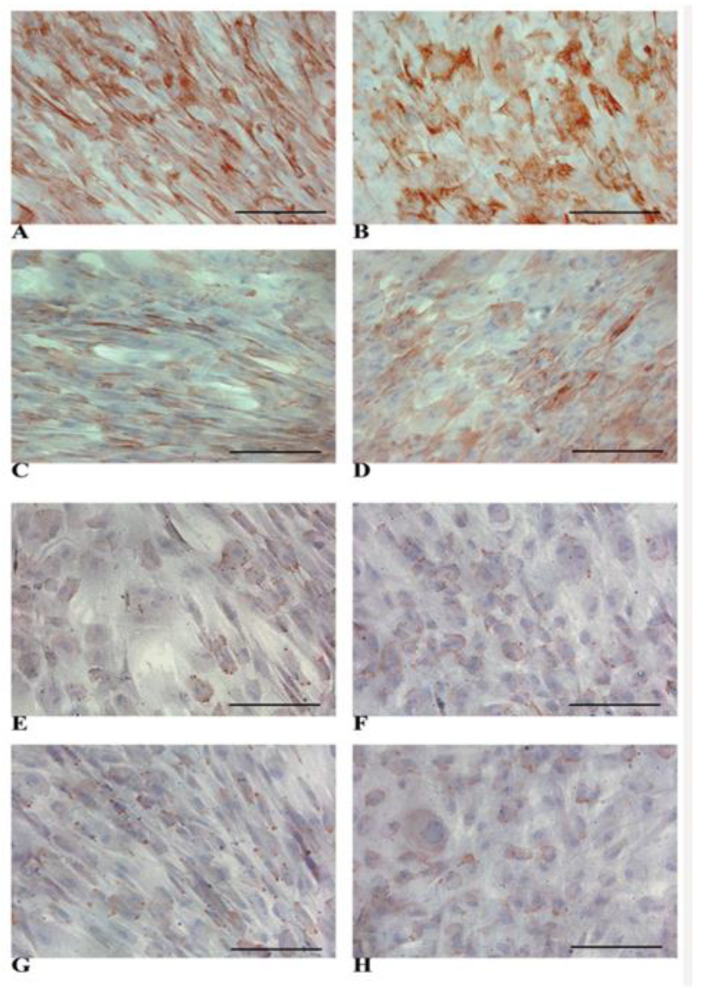
Representative micrographs panel of immunocytochemical detection of markers on NT WJ-MSCs (**A**,**C**) cells and HLCs (**B**,**D**) at all the time points. CK-18 expression is visible in NT WJ-MSCs (**A**,**C**) and HLCs (**B**,**D**), in the 3rd and 4th week of hepatic differentiation. Connexin 32 expression was detectable in NT WJ-MSCs (**E**,**G**) and HLCs (**F**,**H**). (**A**,**B**) and (**E**,**F**): 3rd week; (**C**,**D**) and (**G**,**H**): 4th week. Magnification 20×. Scale bar: 100 µm.

We showed that both NT WJ-MSCs and HLCs exhibited cytoplasmic positivity for CK-18 at the time points considered (Figure 7A–D). A similar pattern was observed for membrane staining of connexin 32 in NT WJ-MSCs and HLCs (Figure 7E–H). In all immunocytochemical analyses, we used an appropriate negative (Figure S3) and positive control (Figure S4). The human liver sections used as positive controls for the immunocytochemical analyses showed a similar expression pattern of liver proteins with respect to the HLCs (Figure S4).

**Figure 8:**
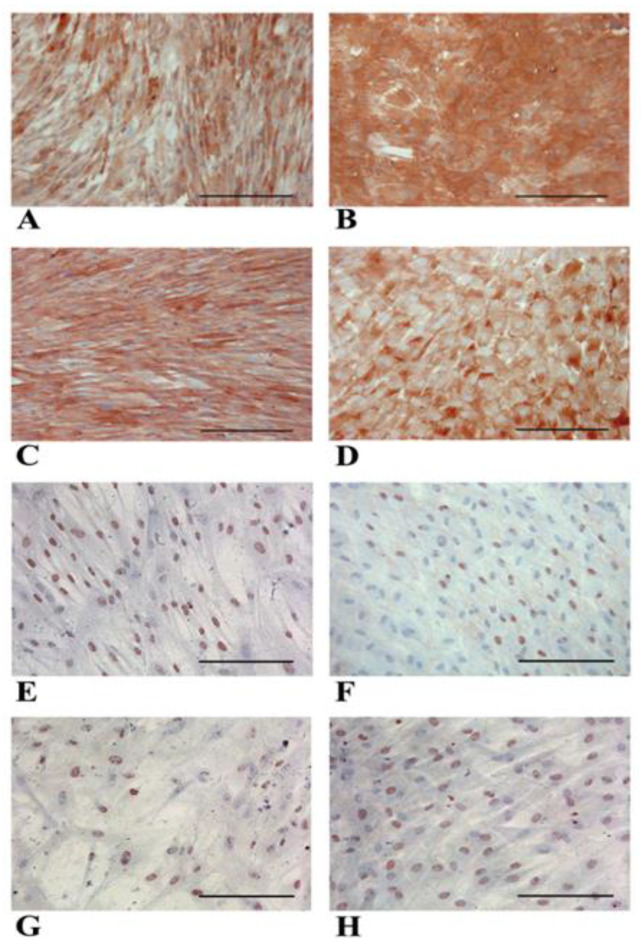
Representative micrographs panel of immunocytochemical detection of albumin in NT WJ-MSCs (**A**,**C**) and HLCs (**B**,**D**), and HNF-4α expression in NT WJ-MSCs (**E**,**G**) and HLCs (**F**,**H)** at 3rd and 4th week of differentiation. (**A**,**B**): 3rd week; (**C**,**D**): 4th week. In NT WJ-MSCs (**E**,**G**). Magnification 20×. Scale bar: 100 µm.

Figure 8 shows albumin expression, which is strongly positive at the cytoplasmic level in both NT WJ-MSCs and HLCs (Figure 8A–D). HNF-4α expression is also maintained at the nuclear level in both NT cells and HLCs. Importantly, we observed that HNF-4α expression and nuclear localization in NT WJ-MSCs decreased with increasing culture time (Figure 8E,G). HLCs maintained the expression and nuclear localization of HNF4α at all time points considered (Figure 8F,H).

**Figure 9:**
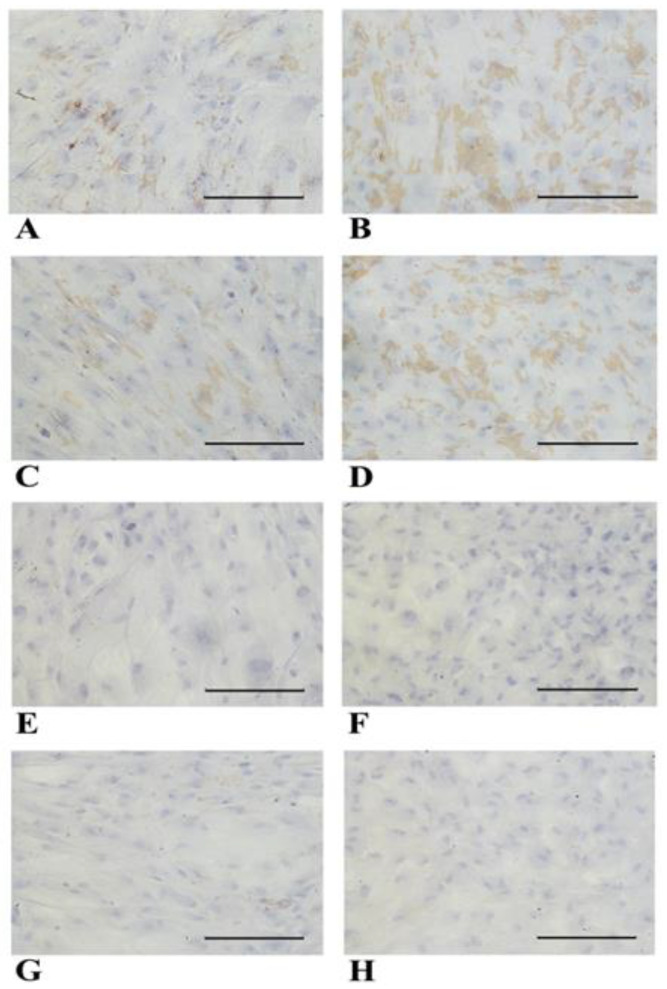
Representative micrographs panel of immunocytochemical detection of fibronectin and collagen IV expression in NT WJ-MSCs and HLCs, at 3rd and 4th week of differentiation. After both third (**B**) and fourth (**D**) week of differentiation, HLCs showed a localization of FN deposits that was markedly altered, being deposited over the surface of HLCs. (**A**,**B)**: 3rd week; (**C**,**D)**: 4th week. A very low expression of collagen IV was detectable in NT WJ-MSCs at both 3rd (**E**) and 4th (**G**) week; HLCs did not express collagen IV at any time point (**F**,**H**). Magnification 20×. Scale bar: 100 µm.

FN is a prominent product of WJ-MSCs and one of the few ECM molecules physiologically produced by hepatocytes in vivo. The morphology of FN extracellular deposits seems to be highly influenced by the applied differentiation protocol: Figure 9A–D shows a clear difference in the morphology of extracellular deposits of HLCs after both the 3rd and 4th week of differentiation steps compared to NT WJ-MSCs.

Conversely, the expression of collagen IV was very low in NT WJ-MSCs at the 3rd (E) and 4th (G) week of differentiation, and the same molecule was not detectable in HLCs at the same time points (Figure 9E–H).

### 3.3. Functional Features Acquired by WJ-MSCs After Hepatic Differentiation

**Figure 10:**
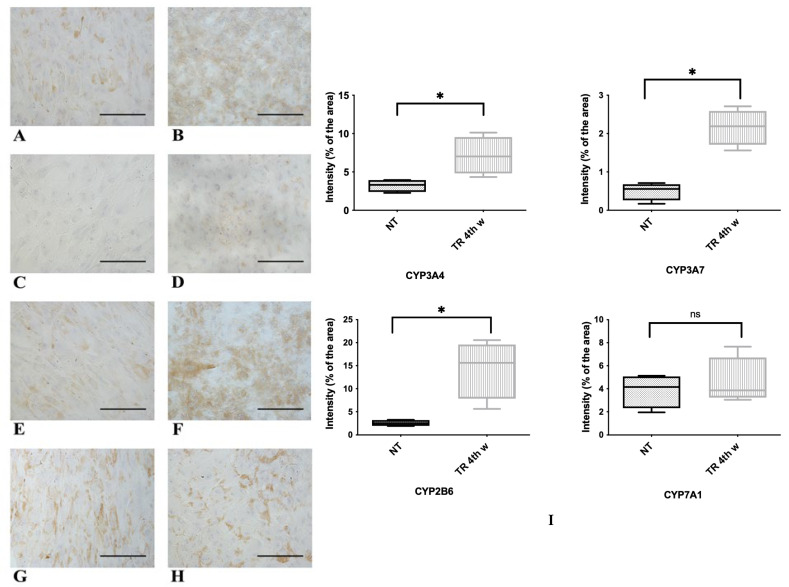
Representative micrographs panel of immunocytochemical detection of different molecules belonging to CYP450s in NT WJ-MSCs (**A**–**G**) and HLCs (here named as TR, (**B**–**H**)), at 4th week of differentiation after rifampicin incubation. The quantifications and statistics are depicted in graph (**I**) with * indicating *p* < 0.05. The statistical analysis was performed by the Mann–Whitney test. CYP3A4 (**A**,**B**), CYP3A7 (**C**,**D**) CYP2B6 (**E**,**F**) were strongly and significantly (**I**) increased in HLCs compared to controls. CYP7A1 was expressed in both NT WJ-MSCs (**G**) and HLCs (**H**) without a significant difference. Magnification 20×. Scale bar: 100 µm.

Hepatocytes carry out important detoxification reactions against xenobiotics through the activity of cytochrome oxidase enzymes known as CYP450 [45]. Since most cytochrome genes are inducible, we examined their expression in both NT WJ-MSCs and HLCs after rifampicin treatment. Figure 10 shows the expression of four CYP450 molecules (namely, CYP3A4, CYP2B6, CYP3A7, and CYP7A1) by ICC. Of the four molecules tested, three, namely, CYP3A4, CYP3A7, and CYP2B6, showed significantly increased positivity in HLCs (Figure 10B,D,F) compared to the controls (NT). In contrast, the expression of CYP7A1 was not significantly different between the two conditions.

**Figure 11:**
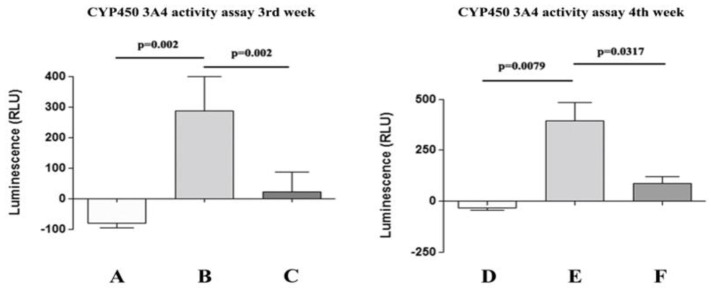
Evaluation of CYP3A4 metabolic activity in NT WJ-MSCs and HLCs at 3rd and 4th week of differentiation. NT WJ-MSCs (**A**,**D**) and HLCs (**B**,**E**) in the presence of rifampicin, an inducer of CYP3A4 activity, despite the being challenged with rifampicin, did not show inducible CYP3A4 activity. HLCs in the presence of ketoconazole, an inhibitor of CYP3A4 activity (**C**,**F**). Statistical analysis was performed by the Mann–Whitney test. (**A**–**C**) are data at the 3rd week of differentiation; (**D**–**F**) are data at the 4th week of hepatic differentiation.

The activity of CYP450 3A4 is widely recognized in the literature as a sign of precursor cells becoming mature hepatocytes, making it one of the most specific CYP450 hepatic isoforms [45]. In NT WJ-MSCs and HLCs, we examined CYP450 3A4 activity in response to a specific inducer (rifampicin), either with or without the addition of an inhibitor (ketoconazole). As shown in Figure 11, NT WJ-MSCs, despite incubation with rifampicin, showed negligible CYP450 3A4 activity levels at both the 3rd (A) and 4th (D) week. On the contrary, HLCs responded to rifampicin, showing a significantly higher CYP450 3A4 activity at all tested time points (Figure 11B,E). Moreover, the specificity of CYP450 3A4 activity was confirmed by a notable decrease in its activity when HLCs were co-incubated with the specific inhibitor ketoconazole (Figure 11C,F). Consistent results were observed at all time points tested. This assay clearly showed that rifampicin induces a significant upregulation of CYP450 3A4 in HLCs (but not in NT WJ-MSCs) and that ketoconazole effectively blocks this induction by specifically inhibiting CYP450 3A4 activity.

**Figure 12:**
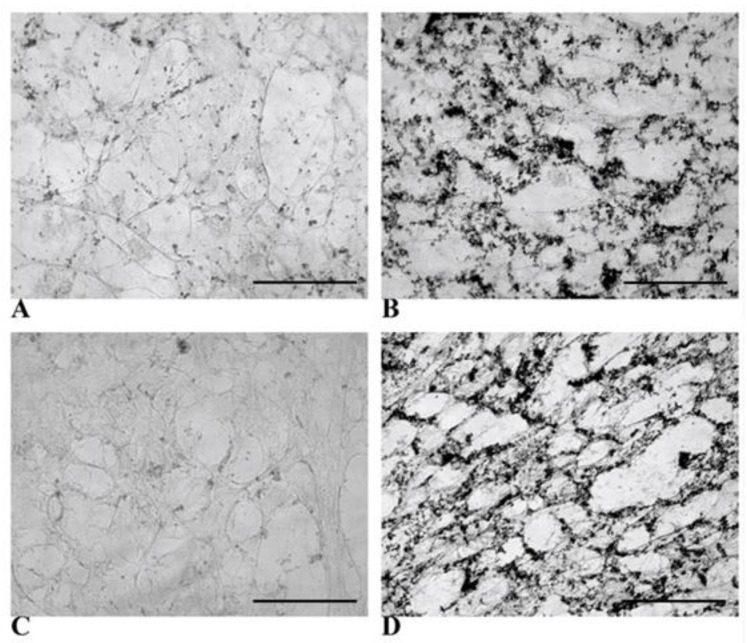
Microscopic demonstration of glucose-6-phosphatase activity in NT WJ-MSCs and HLCs at all considered time points. NT WJ-MSCs (**A**,**C**) and HLCs (**B**,**D**) at 3rd and 4th week of hepatic differentiation; (**A**,**B**): 3rd week; (**C**,**D**): 4th week. Magnification 20×. Scale bar: 100 µm.

The function of glucose-6-phosphatase (G6Pase), which is one of the main enzymes responsible for glucose metabolism in the liver, was evaluated in both control cells and HLCs via another functional assay [37,38]. Figure 12 shows that HLCs exhibited significant brownish–black staining due to the presence of lead sulfide precipitates in both the 3rd and 4th weeks. On the contrary, NT WJ-MSCs appeared negative at both time points.

### 3.4. HLCs Maintain the Immunomodulatory Molecules Responsible for Immune Tolerance

**Figure 13:**
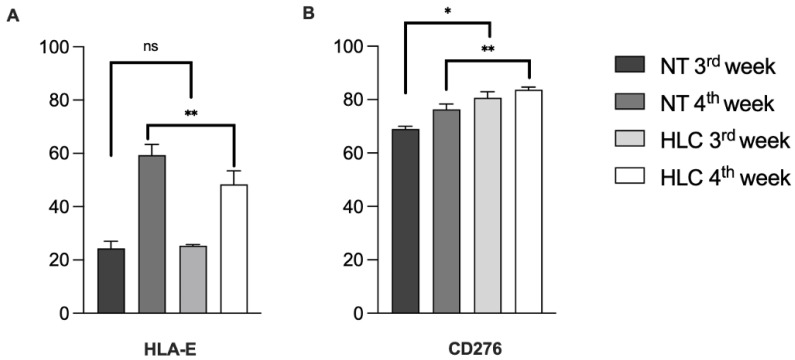
Flow cytometry analysis of immunomodulatory molecules expression levels by NT WJ-MSCs and HLCs at 3rd and 4th week of hepatic differentiation. HLA-E, a non-canonical class I MHC molecule, was expressed in both NT WJ-MSCs and HLCs ((**A**), left panel); also, for CD276 ((**B**), right panel), there was a similar expression with respect to NT cells at each time point. Comparisons among groups were made using Mann–Whitney test. ns, not significant; * *p* ≤ 0.05; ** *p* ≤ 0.01.

We assessed whether HLCs retained the same pattern of immunomodulatory molecule expression previously observed in naïve cells. We had earlier demonstrated that MHC class I (HLA-ABC) expression is preserved after differentiation (Figure 5). Additionally, both NT WJ-MSCs and HLCs exhibit low levels of HLA-DR expression (Figure 5). For the first time, we also showed that both NT WJ-MSCs and HLCs express HLA-E (Figure 13A), a molecule involved in tolerance induction mechanisms in vivo. Representative immunocytochemical analyses of these three HLAs in both NT and HLC cells are shown in Figure S5. B7H3 (also known as CD276) is an immunomodulatory protein from the B7 family recognized for its co-inhibitory role both in vitro and in vivo. We found that both NT WJ-MSCs and HLCs express B7H3, with a slightly higher expression in the differentiated cells compared to the parental cells (Figure 13B).

**Figure 14:**
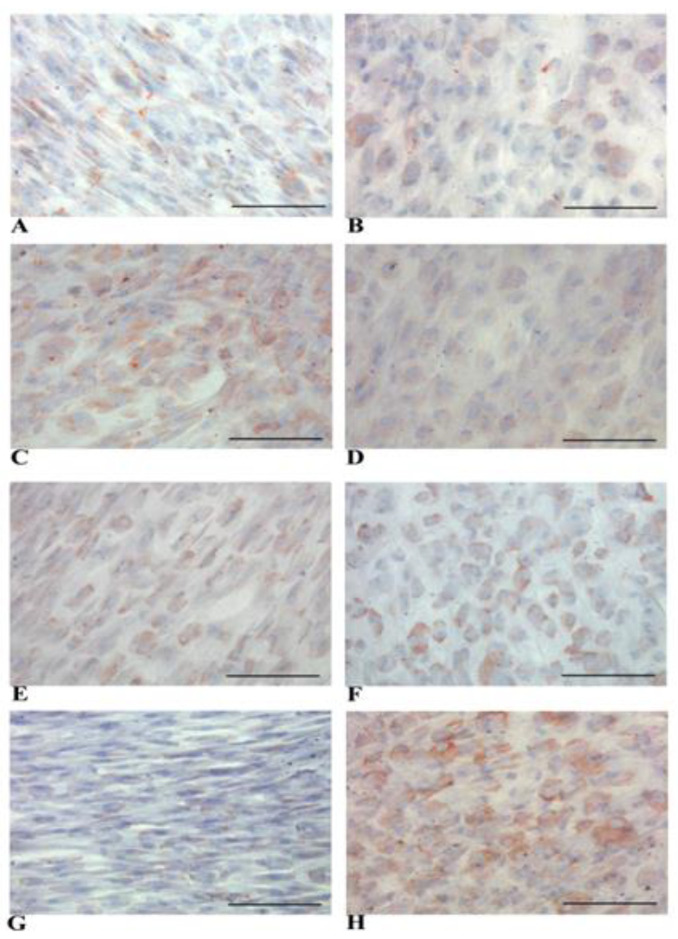
Representative micrographs panel of immunocytochemical detection of B7-H3 (CD276) and IDO-1 in NT WJ-MSCs and HLCs at 3rd and 4th week of hepatic differentiation. CD276 expression in NT WJ-MSCs (**A**,**C**) and HLCs (**B**,**D**), at any time point. IDO-1 expression in NT WJ-MSCs (**E**,**G**) and HLCs (**F**,**H**) at all time points. (**A**,**B)** and (**E**,**F**): 3rd week; (**C**,**D**) and (**G**,**H**): 4th weeks. Magnification 20×. Scale bar: 100 µm.

Immunocytochemical analysis confirmed that B7-H3 expression was similar in both NT WJ-MSCs and HLCs at all time points (Figure 14A–D).

Another proposed mechanism for MSCs’ immune evasion is the secretion of indoleamine 2,3-dioxygenase (IDO-1), an immunomodulatory enzyme involved in tryptophan catabolism, which directly affects lymphocyte proliferation. As shown in Figure 14, we demonstrate for the first time that both NT WJ-MSCs and HLCs express IDO-1, suggesting that the trans-differentiated cells retain this key primitive feature while also acquiring the mature characteristics typical of HLCs (Figure 14E–H).

### 3.5. HPLC-MS/MS Proteome Analyses Identified Novel Intra- and Extracellular Hepatic Proteins in HLCs Derived from WJ-MSCs

**Figure 15:**
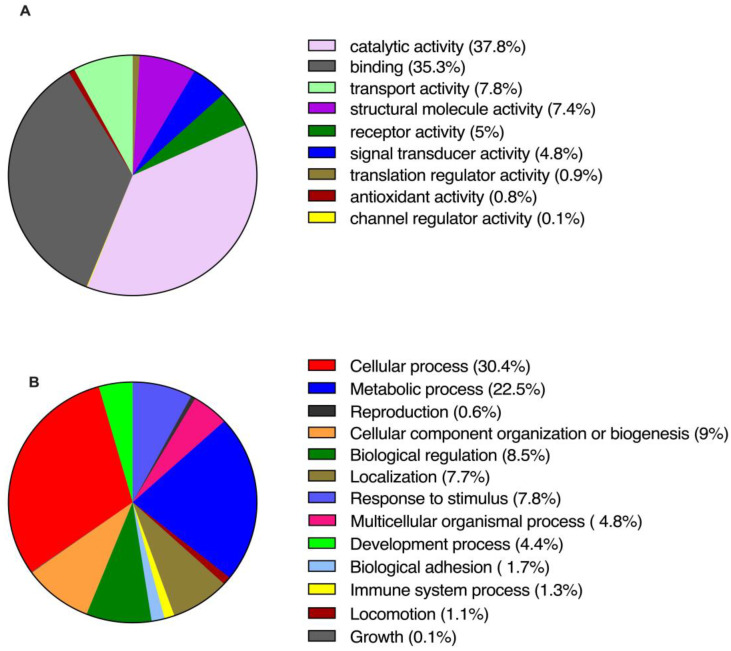
Graphical representation of gene ontology annotations (expressed in percentage of distribution) describing molecular functions (**A**) and biological processes (**B**) of intracellular proteins detected on NT WJ-MSCs and HLCs by HPLC-MS/MS. The pie charts, with related statistics, were automatically, generated by the PANTHER (Protein ANalysis THrough Evolutionary Relationships) GO classification system.

The proteomic analysis enabled the identification of 3643 intracellular proteins. Figure 15 displays the global distribution of these proteins, categorized by molecular function (A) and biological processes (B). The two most prominent functional categories were “binding” and “catalytic activity”, each comprising over 30% of the identified proteins (Figure 15A). In terms of biological processes, most of the proteins were involved in cellular processes (30.4%) and metabolic processes (22.5%). A total of 161 intracellular proteins (listed in Table 1) were chosen among the overall data. We included confirmative MSCs-specific molecules, as well as proteins that are involved in hepatic function, described by means of the other techniques.

Interestingly, we found that WJ-MSCs can express proteins involved in several hepatocyte-specific processes: *AOX1*, *CYP51A1*, *EPHX1*, *FMO3*, *GSTM1*, *GSTO1*, *GSTP1*, *NR1I3*, and *UGT1A4* [46,47,48,49,50,51,52,53].

We found other molecules involved in lipid metabolism, including cholesterol metabolisms: CYP11A1 (mitochondrial form of cholesterol side-chain cleavage enzyme) and CYP39A1 (24-hydroxycholesterol 7-alpha-hydroxylase) [54].

HLCs also expressed molecules such as ATP-binding cassette sub-family A member 1 (ABCA1), the main regulator of HDL and cholesterol export, expressed in the placenta, fetal and adult liver, and other fetal tissues [55,56]; the ATP-binding cassette sub-family A member 6 (ABCA6), highly expressed in the liver [57], and the product of *ABCB4* (phosphatidylcholine translocator ABCB4), which is especially expressed on canalicular membrane of hepatocytes, protecting them from the detergent action exerted by bile salts [58].

Our HLCS expressed, at all time points of differentiation, ACADVL, which encodes for the mitochondrial form of the very long-chain specific acyl-CoA dehydrogenase enzyme, specific to the human liver [59], as well as cytosolic acetyl-CoA acetyltransferase (ACAT2), the exclusive cholesterol-esterifying enzyme in hepatocytes [60], and its mitochondrial form (ACAT1).

The HLCs also featured an enzyme involved in fatty acid metabolism and VLDL biosynthesis, the long-chain-fatty-acid-CoA ligase (ACSL), which was found in two isoforms encoded by *ACSL1* and *ACSL3*, previously described in the liver [61].

We also identified the plasma liver-derived angiopoietin-like protein 3 (ANGPTL3), which plays a role in lipid and lipoprotein metabolism [62], along with the gene products of ECHDC2 and EHHADH, both involved in peroxisomal bile production [63].

The HPLC-MS/MS results also showed the expression of key players in the immune response (Table 1), such as the complement factors C1R, C2, C3, C5, and CFH, all of which are produced by the liver in vivo [64,65,66]; prothrombin (F2), which has a role in acute-phase response [67]; IDO-2, which can be involved in immune modulation exerted by IDO-1 on Tregs generation [68]; galectins 1 and 3 (*LGALS1* and *LGALS3*), which play a role in cell suppression of allogeneic T-cell proliferation and liver regeneration after liver transplantation [69,70]; *MFGE8*, which encodes for a globular protein identified in breast milk (lactadherin) that induces Tregs proliferation and the release of anti-inflammatory IL-10 in newborn’s intestine [71]; *PARK7*, whose gene product is an anti-oxidant protein (DJ-1) that promotes Tregs proliferation and regulates hepatic progenitor cells [72]; PZP, a plasma protein, named pregnancy zone protein, that binds the placental protein-14 on T cell surface to inhibit T cell activation during late pregnancy [73].

#### Quantitative Analysis of Proteins Differentially Expressed in HLCs Revealed an Increase in Liver-Enriched Enzymes with Respect to NT WJ-MSCs

**Figure 16:**
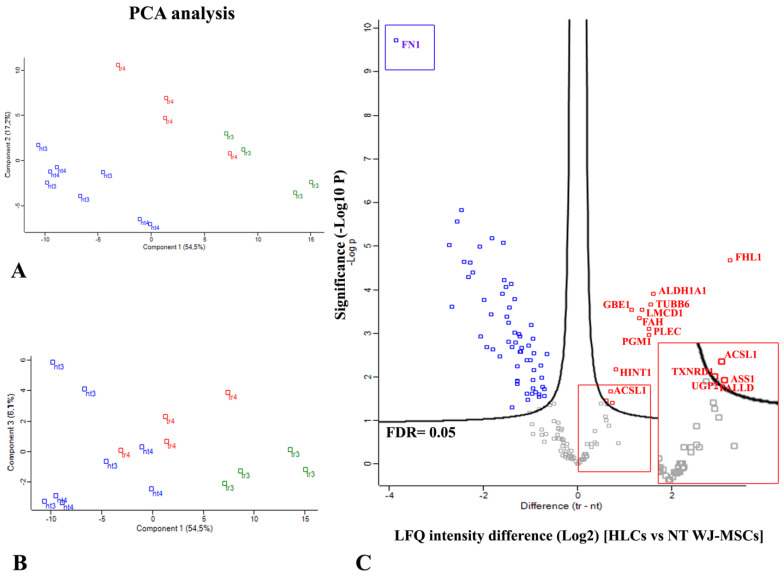
Quantification of differentially expressed proteins using computational analysis of proteomic data. (**A**) Principal component analysis (PCA) between 3rd and 4th week NT WJ-MSCs (blue) and 3rd (green) and 4th (red) week HLCs (tr). (**B**) Graph of PCA considering components 3 and 1 to discriminate 3rd and 4th HLCs; (**C**) Volcano plot showing the significant proteins differentially expressed comparing the differences between HLCs (named as tr) and NT WJ-MSCs (named as nt). The upregulated proteins are shown in red, while downregulated ones are in blue. The 14 upregulated proteins after the differentiation protocol are mentioned in bold red. The insert was used to show some proteins in detail. Fibronectin (FN1, in bold blue) was highly downregulated. X-axis: differences in the intensity of label-free quantification (LFQ) are reported. Y-axis: significance derived from the −log10(*p*-values) of protein ratio between HLCs (tr) and NT WJ-MSCs (nt). FDR = 0.05.

Principal component analysis (PCA) revealed a clear distinction between the intracellular proteins differentially expressed by NT WJ-MSCs (nt) and HLCs (tr), independent of the differentiation week (Figure 16A). Notably, it was possible to discriminate between 3rd- and 4th-week HLCs by plotting the components 3 and 1, while the 3rd and 4th NT WJ-MSCs were not distinguishable (Figure 16B).

The Volcano plot shown in Figure 16C, generated using a *t*-test, highlights significant differences in the expression of intracellular proteins between NT WJ-MSCs and HLCs (see Table S3 for details).

14 proteins were found to be upregulated following the hepatic differentiation protocol, marked in bold red on the right side of the graph. These proteins include FHL1, ALDH1A1, TUBB6, PGM1, PLEC, LMCD1, FAH, GBE1, HINT1, ASS1, ACSL1, PALLD, TXNRD1, and UGP2, as listed in Table S3.

Additionally, no significant differences were observed between the 3rd and 4th week NT WJ-MSCs, while proteins with differential expression were identified in 4th week HLCs compared to those from the 3rd week.

Quantitative analysis revealed a significant increase in the expression of liver-enriched markers in HLCs, such as fumarylacetoacetase (FAH), 1,4-alpha-glucan-branching enzyme (GBE1), long-chain-fatty-acid-CoA ligase 1 (ACSL1), argininosuccinate synthase (ASS1), and UTP-glucose-1-phosphate uridylyltransferase (UGP2), all of which are strongly associated with liver tissue (Table S3). Notably, UGP2, as shown in Table S4, was also significantly more expressed in 4th week HLCs compared to 3rd week HLCs.

Other liver-enriched markers that are upregulated in 4th week HLCs compared to 3rd week HLCs include nicotinamide N-methyltransferase (NNMT), phosphoserine aminotransferase (PSAT1), 7-dehydrocholesterol reductase (DHCR7), and neutral alpha-glucosidase AB (GANAB) (Table S4).

After identifying the 14 most upregulated proteins in HLCs, we utilized the STRING database (https://string-db.org/, accessed on 15 October 2024) to define the known and predicted physical and functional protein–protein interaction networks most associated with liver tissue expression.

**Figure 17:**
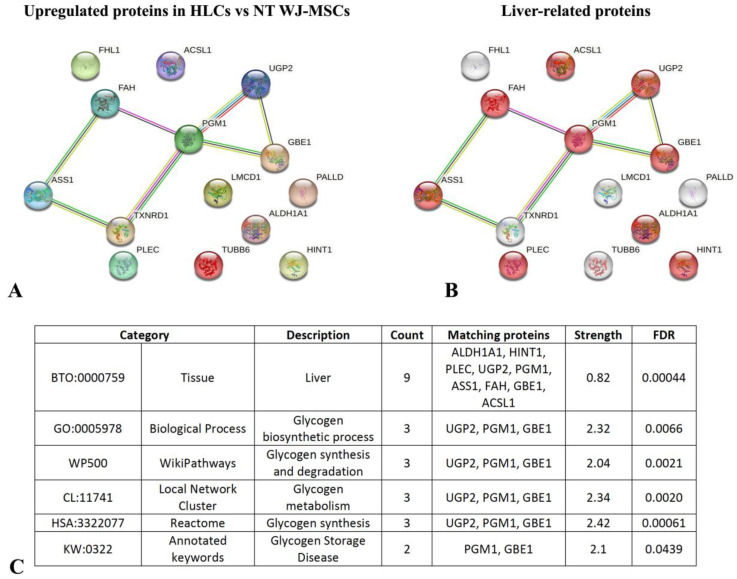
(**A**) STRING interactions of the most upregulated proteins in HLCs after differentiation; (**B**) nine proteins (in red) resulted enriched in liver tissue; (**C**) STRING analysis revealed the proteins strictly connected with some hepatic functions, such as glycogen metabolism.

As shown in Figure 17A, six proteins were found to be interrelated, while nine proteins (Figure 17B) were linked to liver tissue (category BTO:0000759; FDR 0.00044, Table S5). These proteins include retinal dehydrogenase 1 (ALDH1A1), histidine triad nucleotide-binding protein 1 (HINT1), plectin (PLEC), UTP-glucose-1-phosphate uridylyltransferase (UGP2), phosphoglucomutase-1 (PGM1), argininosuccinate synthase (ASS1), fumarylacetoacetase (FAH), 1,4-alpha-glucan-branching enzyme (GBE1), and long-chain-fatty-acid-CoA ligase 1 (ACSL1) (highlighted in red in Figure 17B). Notably, UGP2, as shown in Table S4, is significantly more expressed in 4th week HLCs compared to 3rd week HLCs. Interestingly, three proteins (UGP2, PGM1, GBE1) were related to glycogen metabolism, both synthesis and degradation. Especially GBE1 is known to be related to glycogen storage disease type IV (Figure 17C). More information about the results from the STRING database is listed in Table S5.

## 4. Discussion

Overall, the data presented in this study further support the concept that functional HLCs can be derived from perinatal stromal cells. Stromal cells from perinatal tissues have been proposed to differentiate into HLCs and potentially correct liver diseases, either through a pre-differentiation step or as naïve cells [43,44]. A key feature of perinatal-derived cells is their immunomodulatory ability, which can be maintained even after differentiation, as suggested by previous reports from our group and others [26,74]. Despite the growing body of evidence supporting the use of perinatal cells for HLC derivation, comprehensive information on the overall characteristics and biology of these candidate cells for transplantation is still lacking.

To address this gap, our study focused on an extensive characterization of HLCs derived from WJ-MSCs, analyzing markers, functional activities, and the expression of immunomodulatory molecules. This work aims to broaden the therapeutic potential of these cells for various applications.

Our findings offer mechanistic insights into the results observed from WJ-MSCs transplantation in vivo. The key points from our study are as follows:

(i) WJ-MSCs can be differentiated into HLCs using the same factors involved in hepatocyte specification in vivo. These HLCs exhibit multiple hepatocyte differentiation markers and display several mature hepatocyte functions.

(ii) HLCs retain the expression of molecules that may contribute to their immune privilege even after differentiation.

The comprehensive characterization of HLCs derived from WJ-MSCs, which was further detailed by proteomic analyses, indicated that the differentiation protocol has successfully generated a cellular population showing many features of mature hepatocytes.

For the first time, we have also shown that HLCs acquire specific abilities to secrete components of their pericellular matrix. Although often overlooked, the balance between a “healthy” and “diseased” pericellular matrix is critical for hepatocyte function. Pathological conditions such as liver fibrosis and cirrhosis are characterized by significant changes in the extracellular matrix that affect hepatocyte physiology. We wanted to investigate whether the differentiation protocol affects the secretion of important extracellular matrix components such as fibronectin (FN) and type IV collagen. For the first time, we were able to show that HLCs alter the deposition of fibronectin (FN) in the extracellular space because of the differentiation process. We observed that FN in HLCs is predominantly deposited at the apical membrane of the cells, mimicking the “disrupted basement membrane” typically formed by hepatocytes towards the Disse space. In vivo, this membrane lacks the deposition of collagen IV, a protein that is not produced by hepatocytes. Our results highlighted that collagen IV is deposited at very low levels in naïve WJ-MSCs, and its presence further decreases in HLCs. These data suggest that transplanted HLCs could secrete different factors in loco, restoring the correct combination of components of the extracellular milieu as in healthy liver parenchyma.

Hepatocyte maturation is tightly regulated at the transcriptional level [38]. Therefore, we investigated the expression of a key factor from the FoxA protein family, known as hepatocyte nuclear factor 4 alpha (HNF-4α), which plays a critical role in liver development at both early and late stages. Our data, for the first time, demonstrated that naïve WJ-MSCs specifically express HNF-4α (Figure 2D), suggesting its involvement in the activation of genes such as AFP and albumin, which are early and late hepatocyte-specific markers, respectively. Both genes contain inducible elements in their promoter regions that respond to HNF-4α binding. This finding provides additional support for the use of differentiation protocols aimed at generating HLCs, further enhancing the data from previous studies [41].

Here, we also highlighted that HLCs, apart from featuring several “hepatocyte markers”, should be able to perform the critical physiological functions of hepatocytes such as glucose metabolism and xenobiotics detoxifications. HPLC-MS/MS allowed us to confirm the expression of proteins that were already characterized in this work with other techniques and highlighted several new markers, such as enzymes involved in xenobiotic detoxification, lipid, cholesterol, bile acids, and alcohol metabolisms, in the urea cycle, and in liver morphogenesis and/or regeneration. We identified the product of *AOX1*, a gene that encodes aldehyde oxidase, an enzyme responsible for the oxidation of aldehydes into carboxylic acids. This enzyme plays a role in drug metabolism and hepatic clearance, a feature previously shown to be maintained in cryopreserved hepatocytes [46]. CYP51A1 encodes lanosterol 14-alpha demethylase, which is involved in both xenobiotic metabolism and the biosynthesis of steroids, lipids, and vitamins. This enzyme contributes to the formation of cholesterol and bile acids in the human liver [47]. EPHX1 encodes epoxide hydrolase 1, an enzyme involved in xenobiotic metabolism and bile acid transport into hepatocytes [48].

FMO3 encodes dimethylaniline monooxygenase [N-oxide-forming] 3, an enzyme essential for oxidative drug metabolism, which is expressed in the post-natal liver [49]. GSTM1 encodes glutathione S-transferase class Mu 1, which, along with GSTP1 (glutathione S-transferase class Pi 1), participates in phase II metabolism of xenobiotics. These molecules were also observed in human embryonic stem cell (hESC)-derived HLCs in a study by Söderdahl and colleagues [50].

Furthermore, our experiments revealed that HLCs express glutathione S-transferase class Omega 1 (GSTO1), which is known to be present in human hepatocytes [51], but, to our knowledge, has not been reported in other HLCs. NR1I3, another key protein identified, is involved in xenobiotic metabolism and activates CYP450 enzymes in hepatocytes [52]. *UGT1A4* encodes for the UDP-glucuronosyltransferase 1–4, an enzyme involved in the glucuronidation pathway that transforms small lipophilic molecules, many tertiary amines and drugs, into water-soluble excreted metabolites, and it is normally found in liver microsomes [53]. We also showed the presence of further liver-specific enzymes such as cytoplasmic and mitochondrial aspartate aminotransferase (GOT1 and GOT2), alanine aminotransferase (GPT1), ornithine aminotransferase (OAT), mitochondrial ornithine transporter 1 (SLC25A15), argininosuccinate synthase (ASS1), alcohol dehydrogenase class-3 (ADH5), and the mitochondrial aldehyde dehydrogenase (ALDH2). Their imbalance has also been related to liver diseases [75,76,77].

Based on gene ontology classification, it was possible to ascribe specific functions to the proteins found in our experiments. The quantitative analyses further provided elements supporting these results, also allowing us to characterize the increase in liver-enriched molecules over the weeks of differentiation. This could suggest that WJ-MSCs, following the hepatic differentiation protocol, are not intended to generate mature hepatocytes but may be considerably useful in supporting hepatic functions if transplanted in loco. For example, glycogen storage disease type IV (GSD IV, Andersen’s Disease) is a rare autosomal recessive disorder caused by either GBE deficiency (leading to the manifestation of progressive hepatic fibrosis characterized by hepatomegaly that rapidly progresses to liver cirrhosis and death at early age) or mutation of the same gene inducing a non-progressive hepatic form [78,79]. The statistically significant increase in GBE1 expression in HLCs derived from WJ-MSCs could lead to possible therapeutic use for treating these specific hepatic diseases.

Another potential clinical indication that may come from the present work lies in the activity levels of G6Pase detected in differentiated HLCs. G6Pase dysfunction due to inborn mutations causes glycogen storage disease type Ia (GSD-1a, Von Gierke’s disease). The primary symptom of GSDI in infancy is a low blood sugar level (hypoglycemia). Symptoms of GSDI usually begin from three to four months of age and include enlargement of the liver (hepatomegaly) and kidney (nephromegaly), and elevated levels of lactate, uric acid, and lipids [80,81,82]. The successful induction of G6Pase functional activity in HLCs, with respect to untreated control cells, could open new ways to the use of these cells for treating this genetic disorder currently managed by the evaluation of clinical, laboratory, and imaging data, together with a sole dietary intervention [81].

Our data reinforce the concept that the differentiated progeny of WJ-MSCs maintains the expression of immunomodulatory molecules, which may be beneficial for cellular therapy applications in ESLD. The presence of immunomodulatory activities, which may result in hypoimmunogenicity and tolerance induction, is a key feature of MSCs.

The maintenance of immunomodulatory molecule expression in perinatal-derived HLCs, which we demonstrate here for the first time, may be a crucial characteristic of their therapeutic potential. HLCs, with their immunomodulatory and anti-inflammatory properties, could alter the local microenvironment and stimulate the reactivation of resident progenitor cells, thereby promoting organ self-repair. This, combined with the functional capabilities of HLCs, underscores the potential of human umbilical cord (UC)-derived cells as a safe and versatile source for therapeutic applications. They may not only facilitate a bridging repopulation effect but also exert a paracrine bystander effect to reactivate local progenitor cells suppressed by the underlying disease. A notable example is given by the expression of IDO-2, which, although less studied than IDO-1, is produced by HLCs and has been implicated in immune regulation, particularly within the liver [68].

The plethora of novel markers of the hepatocyte mature phenotype, characterized in our experiments, are indicative of multiple functional competencies and represent a promising advance in the knowledge of the basic biology of these cells, envisioning new therapeutic approaches. Moreover, it may stimulate further research in other laboratories to fully understand the multiple features of HLCs derived from perinatal stromal cells. In this regard, the eventual integration of the proteomics data with transcriptomic ones, as well as the in vivo readout on an animal model, would be necessary developments of the present work and can hopefully stimulate further research on this topic. Moreover, in the future, it would be interesting to investigate the fate of transplanted cells after infusion in an in vivo model. Some reports suggest that these cells could be scavenged by local macrophages [83]. It is important, in in vivo applications, to determine if the potential immunomodulatory features of HLCs are maintained in a compromised microenvironment such as that featured in liver conditions such as cirrhosis and fibrosis. On a positive note, further reports demonstrate that MSCs, after transplantation in vivo, can recreate an immunosuppressive microenvironment, evading the immune response in the host [84,85]. Further in vivo assays would also be beneficial to test other potential key limitations of our approach in the differentiation of HLCs. One specific need of cell-based systems is the scalability of the expansion of stem cells to have the number of differentiated cells needed for therapeutic success. Our previous reports indicate that WJ-MSCs feature a stable phenotype alongside culture up to 15 passages and, therefore, may be particularly promising in this aspect [18]. One other limitation that may arise from the use of in vitro differentiated HLCs is the maintenance of the mature features after infusion in the patient. It must be noted that, often, the mixed result that can be observed in the literature may be linked to the incomplete characterization of the differentiated populations, relying on few markers. The present paper results show that the application of several parallel techniques improves this aspect and allows the characterizing of many potential indications for HLCs based on the selected features detected.

For any differentiation attempt aiming to generate the “perfect hepatocyte”, the only outcome obtainable is a failure. No in vitro differentiation can today mimic the in-organ differentiation that takes place in hepatocytes during liver development. However, this and the other works in the literature [86,87,88,89,90] show that the potential of exogenous stem cells may be to be differentiated to acquire specific liver functions, which allow them to be successful in treating especially monogenetic liver diseases by providing selected activities missing in the patients’ hepatocytes.

## 5. Conclusions

The use of high-throughput techniques, coupled with a detailed morphofunctional evaluation of the differentiated cells phenotype, allows us to determine the potential features of HLCs derived from WJ-MSCs, which show important characteristics potentially useful in liver repopulation strategies for specific diseases and, more generally, in regenerative medicine approaches for ESLD. Even if these data should be further confirmed with WJ-MSCs lots derived from a higher number of umbilical cords, our paper shed light on the important biological properties of WJ-MSCs and their differentiated counterparts. Demonstrating insights into the hepatocyte-related functions exerted by HLCs could be essential to move forward preclinical in vivo experiments and to project clinical trials for the treatment of ESLD.

## Figures and Tables

**Table 1:** Identification of intracellular proteins based on HPLC-MS/MS-derived sequences from NT WJ-MSCs and HLCs. The proteins were classified based on gene ontology annotations and literature references. (*) proteins described for the first time in HLCs derived from WJ-MSCs but already described in HLCs derived from other stem cells. (**) proteins described for the first time in HLCs derived from WJ-MSCs and never described in HLCs derived from other stem cells. (§) proteins expressed by hepatic progenitor cells.

Biological Process	Name	Accession Number	Gene	3rd Week NT WJ-MSCs	3rd Week HLCs	4th Week NT WJ-MSCs	4th Week HLCs
Xenobiotic and drug metabolism process	Aflatoxin B1 aldehyde reductase member 2 (Fragment)	H3BLU7	AKR7A2	−	+	−	+
	Aldehyde oxidase (**)	Q06278	AOX1	−	+	−	−
	Cytochrome P450 1B1	Q16678	CYP1B1	−	−	−	+
	Cytochrome P450 2A13	Q16696	CYP2A13	−	−	−	+
	Cytochrome P450 2S1	Q96SQ9	CYP2S1	+	+	−	−
	Dimethylaniline monooxygenase [N-oxide-forming] 3 (**)	P31513	FMO3	−	−	−	+
	Epoxide hydrolase 1 (*)	P07099	EPHX1	+	+	+	+
	Glutathione S-transferase Mu 1 (**)	P09488	GSTM1	−	−	−	+
	Glutathione S-transferase omega-1 (**)	P78417	GSTO1	+	+	+	+
	Glutathione S-transferase P (**)	P09211	GSTP1	+	+	+	+
	Lanosterol 14-alpha demethylase (**)	Q16850	CYP51A1	−	+	−	+
	Nuclear receptor subfamily 1 group I member 3 (**)	E9PCF2	NR1I3	−	**−**	**−**	+
	UDP-glucuronosyltransferase 1–4 (**)	P22310	UGT1A4	-	+	−	−
Lipid metabolic process	Acetyl-CoA acetyltransferase, cytosolic (**)	Q9BWD1	ACAT2	−	+	**−**	+
	Acetyl-CoA acetyltransferase, mitochondrial	P24752	ACAT1	−	+	**−**	+
	Acyl-coenzyme A thioesterase 2, mitochondrial	P49753	ACOT2	+	+	−	−
	Angiopoietin-related protein 3 (**)	Q9Y5C1	ANGPTL3	−	+	**−**	**−**
	ATP-binding cassette sub-family A member 1 (**) (§)	O95477	ABCA1	−	**−**	**−**	+
	Carboxylesterase 3	Q6UWW8	CES3	−	+	**−**	**−**
	Enoyl-CoA hydratase domain-containing protein 2, mitochondrial (Fragment) (**)	F5GWU3	ECHDC2	−	**−**	**−**	+
	Erlin-1 (**)	O75477	ERLIN1	−	**−**	**−**	+
	Fatty acid synthase	P49327	FASN	−	**−**	**−**	+
	Heme oxygenase 2 (**)	P30519	HMOX2	−	+	−	−
	Hydroxyacyl-coenzyme A dehydrogenase, mitochondrial	Q16836	HADH	−	+	**−**	**−**
	Long-chain-fatty-acid--CoA ligase 1 (**)	E7EPM6	ACSL1	−	+	**−**	+
	Long-chain-fatty-acid--CoA ligase 3 (**)	O95573	ACSL3	−	+	**−**	+
	Mevalonate kinase (**)	F5H8H2	MVK	−	**−**	**−**	+
	Peroxisomal bifunctional enzyme (**)	Q08426	EHHADH	−	**−**	**−**	+
	Phosphatidylcholine translocator ABCB4	P21439	ABCB4	−	+	**−**	**−**
	Very long-chain specific acyl-CoA dehydrogenase, mitochondrial (**)	P49748	ACADVL	−	+	+	+
	Very-long-chain (3R)-3-hydroxyacyl-CoA dehydratase 3 (**)	Q9P035	HACD3	+	**−**	**−**	**+**
	Very long-chain acyl-CoA synthetase	O14975	SLC27A2	−	**−**	**−**	**+**
Cholesterol, bile and carnitine metabolic process	4-trimethylaminobutyraldehyde dehydrogenase (**)	P49189	ALDH9A1	−	+	−	+
	7-dehydrocholesterol reductase (**)	Q9UBM7	DHCR7	+	+	+	+
	Delta (24)-sterol reductase (**)	Q15392	DHCR24	−	+	**−**	+
	24-hydroxycholesterol 7-alpha-hydroxylase (**)	Q9NYL5	CYP39A1	−	+	**−**	**−**
	Cholesterol side-chain cleavage enzyme, mitochondrial	P05108	CYP11A1	−	+	**−**	**−**
	Bile acid receptor (**)	Q96RI1	NR1H4	−	+	**−**	**−**
	Epididymal secretory protein E1 (**)	G3V3E8	NPC2	+	+	+	+
	Leptin receptor	P48357	LEPR	**−**	**−**	−	+
Glucose metabolic process	1,4-alpha-glucan-branching enzyme (**)	Q04446	GBE1	+	+	+	+
	Alpha-enolase	P06733	ENO1	+	+	+	+
	Alpha-N-acetylglucosaminidase	P54802	NAGLU	+	+	−	+
	ATP-dependent 6-phosphofructokinase, liver type (**)	P17858	PFKL	+	+	+	+
	Glycogen debranching enzyme (**)	P35573	AGL	−	+	+	+
	Glycogen phosphorylase, liver form (**)	P06737	PYGL	−	−	−	+
	Glycogen [starch] synthase, muscle	P13807	GYS1	−	+	−	+
	Insulin receptor substrate 2 (**)	Q9Y4H2	IRS2	−	−	−	+
	L-lactate dehydrogenase A chain	P00338	LDHA	+	+	+	+
	Phosphoenolpyruvate carboxykinase [GTP], mitochondrial (**)	Q16822	PCK2	−	+	−	+
	Phosphoglucomutase-1 (**)	P36871	PGM1	+	+	+	+
	Thioredoxin, mitochondrial	Q99757	TXN2	−	−	−	+
	Insulin-like growth factor-binding protein 4	P22692	IGFBP4	−	−	−	+
	Non-POU domain-containing octamer−binding protein	Q15233	NONO	−	**−**	**−**	+
	Protein deglycase DJ-1	Q99497	PARK7	+	+	+	+
	Prothrombin	P00734	F2	+	−	+	+
Amino acid metabolic process	Amine oxidase [flavin-containing] A	P21397	MAOA	−	+	−	+
	Aminopeptidase N (CD13)	P15144	ANPEP	+	+	+	+
	Alanine aminotransferase 1 (**)	P24298	GPT	−	+	−	−
	Aspartate aminotransferase, cytoplasmic (**)	P17174	GOT1	−	+	−	+
	Aspartate aminotransferase, mitochondrial (**)	P00505	GOT2	+	+	+	+
	Carnosine N-methyltransferase	Q8N4J0	CARNMT1	−	−	−	+
	Cathepsin D	P07339	CTSD	−	−	−	+
	Neprilysin (CD10)	P08473	MME	+	+	+	+
	Phosphoserine aminotransferase	Q9Y617	PSAT1	+	+	+	+
	Putative aspartate aminotransferase, cytoplasmic 2	Q8NHS2	GOT1L1	−	−	−	+
Urea cycle	Argininosuccinate synthase (**) (§)	P00966	ASS1	+	+	+	+
	Mitochondrial ornithine transporter 1 (**)	Q9Y619	SLC25A15	−	−	−	+
	Ornithine aminotransferase, mitochondrial (**)	P04181	OAT	+	+	+	+
Alcohol metabolic process	Alcohol dehydrogenase class-3 (**)	P11766	ADH5	+	+	+	+
	Aldehyde dehydrogenase, mitochondrial (**)	P05091	ALDH2	−	+	−	−
Binding	Albumin	P02768	ALB	+	+	+	+
	Apolipoprotein L5	Q9BWW9	APOL5	−	−	−	+
	ATP-binding cassette sub-family A member 6 (**)	Q8N139	ABCA6	−	−	−	+
	CREB-binding protein	Q92793	CREBBP	−	**−**	**−**	+
	Cytochrome b-c1 complex subunit 1, mitochondrial	P31930	UQCRC1	+	+	−	+
	Cytochrome c1, heme protein, mitochondrial (**)	P08574	CYC1	−	+	−	−
	Cytochrome c oxidase subunit 2	P00403	MT-CO2	+	+	−	−
	Cytochrome c oxidase subunit 4 isoform 1, mitochondrial	P13073	COX4I1	+	+	−	+
	Cytochrome c oxidase subunit 5A, mitochondrial	P20674	COX5A	+	+	+	+
	Exportin-T	O43592	XPOT	−	−	−	+
	Filamin-binding LIM protein 1	Q8WUP2	FBLIM1	+	+	−	+
	Hemoglobin subunit gamma-2 (**)	E9PBW4	HBG2	−	+	−	−
	Immediate early response 3-interacting protein 1	Q9Y5U9	IER3IP1	−	−	−	+
	Insulin-like growth factor-binding protein 7 (**)	Q16270	IGFBP7	+	+	+	+
	Metallothionein-2 (**)	P02795	MT2A	−	−	−	+
	Multidrug resistance-associated protein 5	O15440	ABCC5	−	−	−	+
	Poly(rC)-binding protein 1	Q15365	PCBP1	+	+	+	+
	Putative cytochrome b-c1 complex subunit Rieske-like protein 1	P0C7P4	UQCRFS1P1	−	+	−	−
	Transferrin	P02786	TFRC	+	−	−	+
Cellular and apoptotic process	5′-nucleotidase (CD73)	P21589	NT5E	+	+	+	+
	Adipocyte plasma membrane-associated protein	Q9HDC9	APMAP	−	+	−	−
	Carnosine N-methyltransferase	Q8N4J0	CARNMT1	−	−	−	+
	Cytochrome P450 1B1	Q16678	CYP1B1	−	−	−	+
	Death-inducer obliterator 1 (**)	Q9BTC0	DIDO1	−	+	−	−
	Eukaryotic translation initiation factor 3 subunit F	O00303	EIF3F	−	+	−	+
	Gelsolin (**)	P06396	GSN	+	+	−	+
	Inter-alpha-trypsin inhibitor heavy chain H4 (**)	Q14624	ITIH4	−	+	−	−
	mRNA-capping enzyme	O60942	RNGTT	−	−	−	+
	multiple epidermal growth factor-like domains protein 8	Q7Z7M0	MEGF8	+	+	+	+
	PH domain leucine-rich repeat-containing protein phosphatase 1	O60346	PHLPP1	−	−	−	+
	Protein-glutamine gamma-glutamyltransferase 2	P21980	TGM2	+	+	+	+
	Retinoid-inducible serine carboxypeptidase	Q9HB40	SCPEP1	+	−	+	+
	Retinol dehydrogenase 11	Q8TC12	RDH11	−	+	−	+
Proliferation/differentiation and liver development	Dapper homolog 2	Q5SW24	DACT2	−	−	−	+
	Fascin (**)	Q16658	FSCN1	+	+	+	+
	Fibroblast growth factor receptor 2	D2CGD1	FGFR2	−	−	−	+
	Growth/differentiation factor 8	O14793	MSTN	−	−	−	+
	H2.0-like homeobox protein (**)	Q14774	HLX	−	−	−	+
	Hepatocyte growth factor receptor (**) (§)	P08581	MET	−	+	−	−
	Hepatocyte nuclear factor 1-alpha	U3KQS6	HNF1A	−	+	−	−
	Hepatocyte nuclear factor 6	Q9UBC0	ONECUT1	−	+	+	−
	Latent-transforming growth factor beta-binding protein 3 (**) (§)	Q9NS15	LTBP3	+	+	−	−
	Prospero homeobox protein 1 (**) (§)	Q92786	PROX1	−	−	−	+
	Transcription factor SOX-4 (**)	Q06945	SOX4	−	−	−	+
	Transgelin	Q01995	TAGLN	+	+	+	+
Immune system process	Beta-2-microglobulin	P61769	B2M	+	−	+	+
	Complement C1r subcomponent	B4DPQ0	C1R	−	+	−	−
	Complement C2 (Fragment)	H0Y868	C2	−	−	−	+
	Complement C3 (Fragment)	M0R0Q9	C3	−	+	−	−
	Complement C5	P01031	C5	−	−	−	+
	Complement factor H	P08603	CFH	−	+	−	−
	Fibronectin	P02751	FN1	+	+	+	+
	Galectin-1	P09382	LGALS1	+	+	+	+
	Galectin-3	P17931	LGALS3	+	+	−	+
	Guanylate-binding protein 1	P32455	GBP1	−	+	−	+
	HLA class I histocompatibility antigen, A-29 alpha chain	P30512	HLA-A	−	+	−	−
	HLA class I histocompatibility antigen, A-68 alpha chain	P01891	HLA-A	−	+	−	+
	Indoleamine 2,3-dioxygenase 2	Q6ZQW0	IDO2	−	−	−	+
	Indoleamine-pyrrole 2,3-dioxygenase	P14902	IDO1	−	+	−	−
	Kallikrein-2 (Fragment)	A0A075B7A6	KLK2	−	−	−	+
	Non-POU domain-containing octamer-binding protein	Q15233	NONO	−	−	−	+
	Protein deglycase DJ-1	Q99497	PARK7	+	+	+	+
	Prothrombin	P00734	F2	+	−	+	+
Other functions	Actin, cytoplasmic 1	P60709	ACTB	+	+	+	−
	Adenylate cyclase type 4	Q8NFM4	ADCY4	−	+	−	+
	Adenylyl cyclase-associated protein 1	Q01518	CAP1	+	+	+	+
	Amyloid-like protein 2	Q06481	APLP2	−	−	−	+
	Calretinin	P22676	CALB2	−	−	−	+
	Calumenin	O43852	CALU	+	+	+	+
	CD44 antigen	H0YDX6	CD44	+	+	+	+
	Cytokeratin 18	P05783	KRT18	+	+	+	+
	Cytokeratin 19	P08727	KRT19	+	−	+	+
	Cytokeratin 8	P05787	KRT8	+	+	+	+
	Desmin	P17661	DES	−	+	−	+
	Fibrillin-1	P35555	FBN1	−	+	+	−
	Filamin-binding LIM protein 1	Q8WUP2	FBLIM1	+	+	−	+
	Growth arrest-specific protein 2	O43903	GAS2	−	−	−	+
	Growth factor receptor-bound protein 7	Q14451	GRB7	−	−	−	+
	Heat shock-related 70 kDa protein 2	P54652	HSPA2	−	+	−	+
	Integrin beta-1 (CD29)	P05556	ITGB1	+	+	+	+
	Kalirin	O60229	KALRN	−	−	−	+
	Lactadherin	Q08431	MFGE8	+	+	+	+
	Laminin subunit alpha-1	P25391	LAMA1	−	+	−	−
	Lipoma-preferred partner	Q93052	LPP	−	+	−	+
	Plastin-3	P13797	PLS3	+	+	+	+
	Platelet-derived growth factor receptor beta	P09619	PDGFRB	−	+	−	+
	Plectin	Q15149	PLEC	+	+	+	+
	PMS1 protein homolog 1	P54277	PMS1	−	−	−	+
	Pregnancy zone protein	P20742	PZP	−	+	−	−
	Procollagen-lysine,2-oxoglutarate 5-dioxygenase 1	Q02809	PLOD1	+	+	+	+
	Septin-9	Q9UHD8	SEPT9	−	+	−	+
	Sparc	P09486	SPARC	+	+	+	+
	T-complex protein 1 subunit gamma	P49368	CCT3	+	+	−	+
	Tenascin-R	Q92752	TNR	−	−	−	+
	Thy-1 membrane glycoprotein (CD90)	P04216	THY1	+	+	+	+
	UDP-glucose:glycoprotein glucosyltransferase 1	Q9NYU2	UGGT1	−	+	+	+

## Data Availability

The data used to generate this paper are available upon request to the corresponding author.

## Supplementary Materials

The following supporting information can be downloaded at https://www.mdpi.com/article/10.3390/biology14020124/s1: Figure S1: Tri-lineage differentiation ability by. NY WJ-MSCs-MSCs (A, C, E); (B) adipogenic differentiation (oil red O stain); (D) osteogenic differentiation (alizarin red S stain); (F) chondrogenic differentiation (alcian blue stain). Magnification: 10×, Bar: 50 µm (A–D), 5× (E, F), Bar: 100 µm. Figure S2: Expression of cytokeratins after hepatic differentiation. Representative flow cytometry analysis performed on NT WJ-MSCs and HLCs, at 3rd and 4th week of hepatic differentiation protocol: AFP (A), Albumin (B), CK-18 (C) and CK-19 (D). Figure S3: Panel of negative controls in immunocytochemical analyses by omitting the primary antibody: (A) NT 3rd week; (B) HLC 3rd week; (C) NT 4th week; (D) HLC 4th week. Magnification 20×, Bar: 100 µm. Figure S4: Representative panels of immunocytochemical detection of multiple hepatic tissue-specific markers in human liver tissue. Micrographs show the expression of Albumin (A), connexin 32 (B), CK-18 (C), CK-19 (D), CYP7 A1 (E), CYP2 B6 (F), CYP3 A4 (G), CYP3 A7 (H), and Fibronectin (I) at the cytoplasm level. HNF-4α showed a clear nuclear positivity (J). Magnification 20×. Scale bar: 100 µm. Figure S5: Immunocytochemical detection of HLAs in NT-WJ-MSCs and HLCs. HLA-ABC (A–D), HLA-DR (E–H), HLA-E (I–L). Magnification 20×, Bar: 100 µm. Table S1: List of antibodies used for flow cytometry analysis; Table S2: list of antibodies used for immunocytochemistry and immunohistochemistry analysis. Table S3: Proteins differentially expressed in NT WJ-MSCs and HLCs. The differences indicate log2(LFQ Intensity HLCs)/log2(LFQ Intensity NT WJ-MSCs); Table S4: Proteins differentially expressed in 3rd and 4th week HLCs. The differences indicate log2(LFQ Intensity HLCs 4th)/log2(LFQ Intensity HLCs 3rd); Table S5: STRING analysis of proteins which expression is significantly different between HLCs and NT-WJ-MSCs. * FDR = False Discovery Rate. This measure describes how significant the enrichment is. Shown are *p*-values corrected for multiple testing within each category using the Benjamini–Hochberg procedure.
