# Supplementary material for: Characterization and Proteomic Profiling of Hepatocyte-like Cells Derived from Human Wharton’s Jelly Mesenchymal Stromal Cells: De Novo Expression of Liver-Specific Enzymes
Source: Biology (Basel). 2025 Jan 24;14(2):124. doi: 10.3390/biology14020124 (PMC11851833; doi:10.3390/biology14020124)
Supplement: Supplementary file 1 [file biology-14-00124-s001.zip › Table S4.docx]

**Table S4**: Proteins differentially expressed in 4^th^ and 3^rd^ week HLCs. The differences indicate log2(LFQ Intensity _HLCs 4th_)/log2(LFQ Intensity _HLCs 3rd_).

| **ID** | **Protein description** | **Gene** | **-log(p-values)** | **Difference** |
| --- | --- | --- | --- | --- |
| P05783 | Keratin, type I cytoskeletal 18 | KRT18 | 3.20 | 2.76 |
| P08133 | Annexin A6 | ANXA6 | 4.09 | 2.50 |
| P07602 | Prosaposin | PSAP | 2.77 | 2.43 |
| O43776 | Asparagine--tRNA ligase, cytoplasmic | NARS | 2.64 | 2.12 |
| P30086 | Phosphatidylethanolamine-binding protein 1 | PEBP1 | 2.04 | 2.04 |
| P10619 | Lysosomal protective protein | CTSA | 1.95 | 1.98 |
| P30041 | Peroxiredoxin-6 | PRDX6 | 3.33 | 1.96 |
| P07858 | Cathepsin B | CTSB | 3.02 | 1.69 |
| Q15293 | Reticulocalbin-1 | RCN1 | 1.34 | 1.63 |
| P07195 | L-lactate dehydrogenase B chain | LDHB | 2.38 | 1.61 |
| P06733 | Alpha-enolase | ENO1 | 2.61 | 1.57 |
| O00764 | Pyridoxal kinase | PDXK | 1.58 | 1.56 |
| P50995 | Annexin A11 | ANXA11 | 1.87 | 1.53 |
| P37837 | Transaldolase | TALDO1 | 1.79 | 1.51 |
| P35754 | Glutaredoxin-1 | GLRX | 1.71 | 1.51 |
| Q969H8 | Myeloid-derived growth factor | MYDGF | 2.32 | 1.49 |
| P09651 | Heterogeneous nuclear ribonucleoprotein A1 | HNRNPA1 | 1.59 | 1.46 |
| P04216 | Thy-1 membrane glycoprotein | THY1 | 2.36 | 1.44 |
| P13667 | Protein disulfide-isomerase A4 | PDIA4 | 2.22 | 1.43 |
| P10253 | Lysosomal alpha-glucosidase | GAA | 2.24 | 1.34 |
| P00338 | L-lactate dehydrogenase A chain | LDHA | 2.16 | 1.34 |
| O00469 | Procollagen-lysine,2-oxoglutarate 5-dioxygenase 2 | PLOD2 | 1.69 | 1.32 |
| Q5EB52 | Mesoderm-specific transcript homolog protein | MEST | 1.56 | 1.31 |
| Q70UQ0 | Inhibitor of nuclear factor kappa-B kinase-interacting protein | IKBIP | 1.47 | 1.28 |
| P21980 | Protein-glutamine gamma-glutamyltransferase 2 | TGM2 | 2.25 | 1.28 |
| Q32P28 | Prolyl 3-hydroxylase 1 | LEPRE1 | 1.80 | 1.27 |
| P30101 | Protein disulfide-isomerase A3 | PDIA3 | 1.93 | 1.25 |
| P00387 | NADH-cytochrome b5 reductase 3 | CYB5R3 | 1.71 | 1.22 |
| P30044 | Peroxiredoxin-5, mitochondrial | PRDX5 | 1.43 | 1.21 |
| P48163 | NADP-dependent malic enzyme | ME1 | 1.76 | 1.21 |
| Q14956 | Transmembrane glycoprotein NMB | GPNMB | 1.35 | 1.20 |
| P29401 | Transketolase | TKT | 1.80 | 1.20 |
| P11021 | 78 kDa glucose-regulated protein | HSPA5 | 1.72 | 1.20 |
| P31949 | Protein S100-A11 | S100A11 | 1.86 | 1.18 |
| P35580 | Myosin-10 | MYH10 | 1.52 | 1.17 |
| P40261 | Nicotinamide N-methyltransferase | NNMT | 2.10 | 1.14 |
| Q14192 | Four and a half LIM domains protein 2 | FHL2 | 1.92 | 1.14 |
| P08670 | Vimentin | VIM | 1.70 | 1.13 |
| P04083 | Annexin A1 | ANXA1 | 1.90 | 1.13 |
| P35579 | Myosin-9 | MYH9 | 1.99 | 1.10 |
| P04075 | Fructose-bisphosphate aldolase A | ALDOA | 1.66 | 1.08 |
| Q9NZU5 | LIM and cysteine-rich domains protein 1 | LMCD1 | 1.55 | 1.07 |
| Q9Y617 | Phosphoserine aminotransferase | PSAT1 | 1.39 | 1.06 |
| P32119 | Peroxiredoxin-2 | PRDX2 | 2.16 | 1.06 |
| P09382 | Galectin-1 | LGALS1 | 1.97 | 1.05 |
| P60175 | Triosephosphate isomerase | TPI1 | 1.80 | 1.05 |
| P07737 | Profilin-1 | PFN1 | 1.53 | 1.04 |
| P36871 | Phosphoglucomutase-1 | PGM1 | 1.53 | 1.04 |
| P49588 | Alanine--tRNA ligase, cytoplasmic | AARS | 1.99 | 1.02 |
| P06865 | Beta-hexosaminidase subunit alpha | HEXA | 2.41 | 1.01 |
| P49327 | Fatty acid synthase | FASN | 1.54 | 1.00 |
| Q15149 | Plectin | PLEC | 1.31 | 0.97 |
| Q8NBS9 | Thioredoxin domain-containing protein 5 | TXNDC5 | 1.64 | 0.96 |
| P04844 | Dolichyl-diphosphooligosaccharide--protein glycosyltransferase subunit 2 | RPN2 | 1.74 | 0.96 |
| Q9UBM7 | 7-dehydrocholesterol reductase | DHCR7 | 1.38 | 0.95 |
| Q15942 | Zyxin | ZYX | 1.26 | 0.95 |
| P07686 | Beta-hexosaminidase subunit beta | HEXB | 1.48 | 0.94 |
| Q13642 | Four and a half LIM domains protein 1 | FHL1 | 1.93 | 0.94 |
| P16278 | Beta-galactosidase | GLB1 | 1.48 | 0.93 |
| P17655 | Calpain-2 catalytic subunit | CAPN2 | 1.64 | 0.92 |
| P06744 | Glucose-6-phosphate isomerase | GPI | 1.32 | 0.91 |
| Q01853 | Transitional endoplasmic reticulum ATPase | Vcp | 1.34 | 0.91 |
| P11766 | Alcohol dehydrogenase class-3 | ADH5 | 1.36 | 0.90 |
| P36542 | ATP synthase subunit gamma, mitochondrial | ATP5C1 | 1.98 | 0.88 |
| P31939 | Bifunctional purine biosynthesis protein PURH | ATIC | 1.33 | 0.87 |
| Q8WX93 | Palladin | PALLD | 1.29 | 0.83 |
| P04406 | Glyceraldehyde-3-phosphate dehydrogenase | GAPDH | 1.33 | 0.82 |
| P41250 | Glycine--tRNA ligase | GARS | 1.29 | 0.82 |
| P39656 | Dolichyl-diphosphooligosaccharide--protein glycosyltransferase 48 kDa subunit | DDOST | 1.39 | 0.81 |
| P51688 | N-sulphoglucosamine sulphohydrolase | SGSH | 1.27 | 0.80 |
| P25705 | ATP synthase subunit alpha, mitochondrial | ATP5A1 | 1.29 | 0.79 |
| Q14697 | Neutral alpha-glucosidase AB | GANAB | 1.23 | 0.75 |
| Q16851 | UTP--glucose-1-phosphate uridylyltransferase | UGP2 | 1.31 | 0.67 |
| Q9BUF5 | Tubulin beta-6 chain | TUBB6 | 1.62 | -0.93 |
| P27487 | Dipeptidyl peptidase 4 | DPP4 | 2.29 | -1.34 |
