# Supplementary material for: Characterization and Proteomic Profiling of Hepatocyte-like Cells Derived from Human Wharton’s Jelly Mesenchymal Stromal Cells: De Novo Expression of Liver-Specific Enzymes
Source: Biology (Basel). 2025 Jan 24;14(2):124. doi: 10.3390/biology14020124 (PMC11851833; doi:10.3390/biology14020124)
Supplement: Supplementary file 1 [file biology-14-00124-s001.zip › Table S3.docx]

**Table S3**:Proteins differentially expressed in HLCs and NT WJ-MSCs. The differences indicate log2(LFQ Intensity _HLCs_)/log2(LFQ Intensity _NT WJ-MSCs_).

| **ID** | **Protein description** | **Gene** | **-log(p-value)** | **Differences** |
| --- | --- | --- | --- | --- |
| Q13642 | Four and a half LIM domains protein 1 | FHL1 | 4.67 | 3.23 |
| P00352 | Retinal dehydrogenase 1 | ALDH1A1 | 3.90 | 1.59 |
| Q9BUF5 | Tubulin beta-6 chain | TUBB6 | 3.66 | 1.55 |
| P36871 | Phosphoglucomutase-1 | PGM1 | 2.96 | 1.51 |
| Q15149 | Plectin | PLEC | 3.10 | 1.51 |
| Q9NZU5 | LIM and cysteine-rich domains protein 1 | LMCD1 | 3.53 | 1.36 |
| P16930 | Fumarylacetoacetase | FAH | 3.34 | 1.30 |
| Q04446 | 1,4-alpha-glucan-branching enzyme | GBE1 | 3.54 | 1.15 |
| P49773 | Histidine triad nucleotide-binding protein 1 | HINT1 | 2.18 | 0.80 |
| P00966 | Argininosuccinate synthase | ASS1 | 1.41 | 0.74 |
| P33121 | Long-chain-fatty-acid--CoA ligase 1 | ACSL1 | 1.66 | 0.69 |
| Q8WX93 | Palladin | PALLD | 1.38 | 0.61 |
| Q16881 | Thioredoxin reductase 1, cytoplasmic | TXNRD1 | 1.45 | 0.60 |
| Q16851 | UTP--glucose-1-phosphate uridylyltransferase | UGP2 | 1.39 | 0.50 |
| Q9BS26 | Endoplasmic reticulum resident protein 44 | ERP44 | 1.38 | -0.50 |
| P40261 | Nicotinamide N-methyltransferase | NNMT | 1.35 | -0.60 |
| Q08431 | Lactadherin | MFGE8 | 2.53 | -0.65 |
| P48047 | ATP synthase subunit O, mitochondrial | ATP5O | 1.56 | -0.70 |
| Q14697 | Neutral alpha-glucosidase AB | GANAB | 1.72 | -0.71 |
| P60033 | CD81 antigen | CD81 | 1.76 | -0.74 |
| P04844 | Dolichyl-diphosphooligosaccharide--protein glycosyltransferase subunit 2 | RPN2 | 1.98 | -0.76 |
| O95340 | Bifunctional 3-phosphoadenosine 5-phosphosulfate synthase 2 | PAPSS2 | 2.26 | -0.79 |
| P27487 | Dipeptidyl peptidase 4 | DPP4 | 1.54 | -0.82 |
| P21589 | 5-nucleotidase | NT5E | 1.60 | -0.83 |
| Q32P28 | Prolyl 3-hydroxylase 1 | LEPRE1 | 1.65 | -0.89 |
| P02461 | Collagen alpha-1(III) chain | COL3A1 | 2.53 | -0.92 |
| P32119 | Peroxiredoxin-2 | PRDX2 | 2.15 | -0.93 |
| P27824 | Calnexin | CANX | 1.93 | -0.95 |
| P06733 | Alpha-enolase | ENO1 | 1.74 | -0.95 |
| P09211 | Glutathione S-transferase P | GSTP1 | 2.88 | -0.95 |
| P37837 | Transaldolase | TALDO1 | 1.62 | -0.97 |
| P08473 | Neprilysin | MME | 3.19 | -0.99 |
| P31949 | Protein S100-A11 | S100A11 | 2.38 | -1.01 |
| Q9NZM1 | Myoferlin | MYOF | 2.72 | -1.04 |
| P07686 | Beta-hexosaminidase subunit beta | HEXB | 1.47 | -1.06 |
| P06865 | Beta-hexosaminidase subunit alpha | HEXA | 2.39 | -1.08 |
| Q70UQ0 | Inhibitor of nuclear factor kappa-B kinase-interacting protein | IKBIP | 1.61 | -1.08 |
| P07737 | Profilin-1 | PFN1 | 2.67 | -1.19 |
| Q14192 | Four and a half LIM domains protein 2 | FHL2 | 2.57 | -1.20 |
| P19367 | Hexokinase-1 | HK1 | 2.58 | -1.23 |
| P04075 | Fructose-bisphosphate aldolase A | ALDOA | 2.93 | -1.23 |
| P39656 | Dolichyl-diphosphooligosaccharide--protein glycosyltransferase 48 kDa subunit | DDOST | 2.97 | -1.25 |
| P09651 | Heterogeneous nuclear ribonucleoprotein A1 | HNRNPA1 | 1.84 | -1.28 |
| P49411 | Elongation factor Tu, mitochondrial | TUFM | 1.89 | -1.28 |
| P30041 | Peroxiredoxin-6 | PRDX6 | 1.91 | -1.29 |
| P04216 | Thy-1 membrane glycoprotein | THY1 | 2.25 | -1.29 |
| Q9UHG3 | Prenylcysteine oxidase 1 | PCYOX1 | 2.79 | -1.32 |
| P35579 | Myosin-9 | MYH9 | 3.01 | -1.34 |
| P16278 | Beta-galactosidase | GLB1 | 3.78 | -1.34 |
| P07858 | Cathepsin B | CTSB | 2.68 | -1.40 |
| Q96HC4 | PDZ and LIM domain protein 5 | PDLIM5 | 1.30 | -1.40 |
| Q07065 | Cytoskeleton-associated protein 4 | CKAP4 | 4.13 | -1.42 |
| P09382 | Galectin-1 | LGALS1 | 3.60 | -1.45 |
| P49257 | Protein ERGIC-53 | LMAN1 | 2.80 | -1.47 |
| P35580 | Myosin-10 | MYH10 | 3.23 | -1.47 |
| P11021 | 78 kDa glucose-regulated protein | HSPA5 | 3.38 | -1.50 |
| P08195 | 4F2 cell-surface antigen heavy chain | SLC3A2 | 4.06 | -1.52 |
| P04179 | Superoxide dismutase [Mn], mitochondrial | SOD2 | 4.22 | -1.56 |
| P07339 | Cathepsin D | CTSD | 5.07 | -1.57 |
| P30101 | Protein disulfide-isomerase A3 | PDIA3 | 3.90 | -1.60 |
| P10619 | Lysosomal protective protein | CTSA | 2.47 | -1.66 |
| P30044 | Peroxiredoxin-5, mitochondrial | PRDX5 | 2.63 | -1.80 |
| P04062 | Glucosylceramidase | GBA | 5.18 | -1.81 |
| Q5EB52 | Mesoderm-specific transcript homolog protein | MEST | 3.43 | -1.84 |
| Q969H8 | Myeloid-derived growth factor | MYDGF | 2.67 | -1.93 |
| Q14956 | Transmembrane glycoprotein NMB | GPNMB | 3.77 | -1.98 |
| P11279 | Lysosome-associated membrane glycoprotein 1 | LAMP1 | 2.92 | -2.06 |
| P21980 | Protein-glutamine gamma-glutamyltransferase 2 | TGM2 | 4.98 | -2.08 |
| O00469 | Procollagen-lysine,2-oxoglutarate 5-dioxygenase 2 | PLOD2 | 4.40 | -2.22 |
| Q8IV08 | Phospholipase D3 | PLD3 | 4.63 | -2.29 |
| P13667 | Protein disulfide-isomerase A4 | PDIA4 | 4.30 | -2.31 |
| P53690 | Matrix metalloproteinase-14 | Mmp14 | 4.64 | -2.40 |
| P07093 | Glia-derived nexin | SERPINE2 | 5.83 | -2.47 |
| Q12884 | Prolyl endopeptidase FAP | FAP | 5.56 | -2.55 |
| P07602 | Prosaposin | PSAP | 3.61 | -2.67 |
| Q14108 | Lysosome membrane protein 2 | SCARB2 | 5.02 | -2.72 |
| P02751 | Fibronectin | FN1 | 9.73 | -3.85 |
