# Supplementary material for: Characterization and Proteomic Profiling of Hepatocyte-like Cells Derived from Human Wharton’s Jelly Mesenchymal Stromal Cells: De Novo Expression of Liver-Specific Enzymes
Source: Biology (Basel). 2025 Jan 24;14(2):124. doi: 10.3390/biology14020124 (PMC11851833; doi:10.3390/biology14020124)
Supplement: Supplementary file 1 [file biology-14-00124-s001.zip › Table S2.docx]

Supplementary Table 2: list of antibodies used for immunocytochemistry and immunohistochemistry (*) analyses.

| **Antigen** | **Clone** | **Host** | **Manufacturer** | **Diluition** |
| --- | --- | --- | --- | --- |
| Albumin (*) | HSA-11 | Mouse monoclonal | Sigma | 1.300 |
| B7H3 (CD276) | H-300 | Rabbit polyclonal | Santa Cruz | 1:100 |
| CYP3A4 (*) | HL3 | Mouse monoclonal | Santa Cruz | 1:100 |
| CYP3A7 (*) | F19-P2-H2 | Mouse monoclonal | Santa Cruz | 1:100 |
| CYP2B6 (*) | H-110 | Rabbit polyclonal | Santa Cruz | 1:200 |
| CYP7A1(*) | H-58 | Rabbit polyclonal | Santa Cruz | 1:100 |
| Collagen IV | COL-94 | Mouse monoclonal | Sigma | 1:300 |
| Connexin 32 (*) | HAM8 | Mouse monoclonal | Santa Cruz | 1:100 |
| Connexin 43 | H-150 | Rabbit polyclonal | Santa Cruz | 1:100 |
| Fibronectin (*) | FN-3E2 | Mouse monoclonal | Sigma | 1:400 |
| HNF 4α (*) | EPR3648 | Rabbit monoclonal | Epitomics | 1:100 |
| IDO | H-11 | Mouse monoclonal | Santa Cruz | 1:50 |
| CK-18 (*) | CY-90 | Mouse monoclonal | Sigma | 1:100 |
| CK-19 (*) | MAB3238 | Mouse monoclonal | Chemicon | 1:100 |
