# Supplementary material for: Characterization and Proteomic Profiling of Hepatocyte-like Cells Derived from Human Wharton’s Jelly Mesenchymal Stromal Cells: De Novo Expression of Liver-Specific Enzymes
Source: Biology (Basel). 2025 Jan 24;14(2):124. doi: 10.3390/biology14020124 (PMC11851833; doi:10.3390/biology14020124)
Supplement: Supplementary file 1 [file biology-14-00124-s001.zip › Table S1.docx]

Supplementary Table 1: list of antibodies used for flow cytometry analysis.

| Antigen | Clone | Conjugated | Dilution | Manufacturer |
| --- | --- | --- | --- | --- |
| IgG1 | G18-145 | FITC | undiluited | Becton Dickinson Biosciences, San Jose, CA |
| IgG2 | PC10 | PE | undiluited | Becton Dickinson |
| albumin | 188835 | unconjugated | 1:20 | R&D Systems |
| AFP | 189506 | unconjugated | 1:40 | R&D Systems, Minneapolis, MN |
| CD29 | mar-04 | APC | undiluited | Becton Dickinson |
| CD31 | WM59 | FITC | undiluited | Becton Dickinson |
| CD34 | 581 | FITC | undiluited | Becton Dickinson |
| CD45 | 2D1 | PerCP | undiluited | Becton Dickinson |
| CD73 | AD2 | APC | 1:11 | Miltenyi Biotec GmbH, Bergisch Gladbach, DE |
| CD90 (Thy-1) | 5 E10 | FITC | 1:20 | Becton Dickinson |
| CD105 | SN6 | FITC | undiluited | Abcam, Cambridge, MA |
| CD117 | YB5.B8 | PE | undiluited | Becton Dickinson |
| CD276 | FM276 | APC | 1:11 | Miltenyi Biotec GmbH |
| CK-18 | C-04 | FITC | 1:20 | Abcam, Cambridge, MA |
| CK-19 | RCK108 | PE | 1:20 | Santa Cruz Biotechnology, Santa Cruz, CA |
| HLA-ABC | W6/32 | FITC | 1:300 | Abcam Cambridge, MA |
| HLA-DR | L243 (G46-6) | PerCP | undiluited | Becton Dickinson |
| HLA-G | 87G | PerCP | undiluited | eBioscience Inc., San Diego, CA |
| HLA-E | 3D12HLA-E | APC | undiluited | eBioscience |
