# Supplementary material for: Characterization and Proteomic Profiling of Hepatocyte-like Cells Derived from Human Wharton’s Jelly Mesenchymal Stromal Cells: De Novo Expression of Liver-Specific Enzymes
Source: Biology (Basel). 2025 Jan 24;14(2):124. doi: 10.3390/biology14020124 (PMC11851833; doi:10.3390/biology14020124)
Supplement: Supplementary file 1 [file biology-14-00124-s001.zip › Table S5.docx]

| **#category** | **term ID** | **term description** | **matching proteins in your network (labels)** | **FDR** |
| --- | --- | --- | --- | --- |
| GO Process | GO:0005978 | Glycogen biosynthetic process | UGP2, PGM1, GBE1 | 0.0066 |
| GO Process | GO:0006091 | Generation of precursor metabolites and energy | ALDH1A1, UGP2, PGM1, GBE1, TXNRD1 | 0.0174 |
| GO Process | GO:0034201 | Response to oleic acid | ASS1, ACSL1 | 0.0174 |
| GO Process | GO:0044281 | Small molecule metabolic process | ALDH1A1, HINT1, UGP2, PGM1, ASS1, FAH, ACSL1, TXNRD1 | 0.0174 |
| GO Process | GO:0055086 | Nucleobase-containing small molecule metabolic process | HINT1, UGP2, PGM1, ACSL1, TXNRD1 | 0.0196 |
| GO Component | GO:0070062 | Extracellular exosome | ALDH1A1, HINT1, TUBB6, PLEC, UGP2, PGM1, ASS1, FAH, GBE1, TXNRD1 | 0.00023 |
| GO Component | GO:0005829 | Cytosol | ALDH1A1, HINT1, PLEC, UGP2, PGM1, ASS1, FHL1, FAH, GBE1, PALLD, TXNRD1 | 0.0208 |
| STRING clusters | CL:11741 | Glycogen metabolism | UGP2, PGM1, GBE1 | 0.0022 |
| STRING clusters | CL:11744 | Carbohydrate phosphorylase, and phosphoglucomutase activity | UGP2, PGM1 | 0.0258 |
| Reactome | HSA-3322077 | Glycogen synthesis | UGP2, PGM1, GBE1 | 0.00061 |
| Reactome | HSA-1430728 | Metabolism | ALDH1A1, UGP2, PGM1, ASS1, FAH, GBE1, ACSL1, TXNRD1 | 0.0205 |
| Reactome | HSA-71387 | Metabolism of carbohydrates | ALDH1A1, UGP2, PGM1, GBE1 | 0.0247 |
| WikiPathways | WP500 | Glycogen synthesis and degradation | UGP2, PGM1, GBE1 | 0.0021 |
| WikiPathways | WP3925 | Amino acid metabolism | ALDH1A1, ASS1, FAH | 0.0127 |
| WikiPathways | WP698 | Glucuronidation | UGP2, PGM1 | 0.0401 |
| Monarch | HP:0001771 | Achilles tendon contracture | HINT1, PLEC, FHL1 | 0.0260 |
| TISSUES | BTO:0000083 | Female reproductive system | LMCD1, ALDH1A1, HINT1, TUBB6, PLEC, UGP2, PGM1, ASS1, FHL1, FAH, GBE1, ACSL1, PALLD, TXNRD1 | 7.8e-05 |
| TISSUES | BTO:0000759 | Liver | ALDH1A1, HINT1, PLEC, UGP2, PGM1, ASS1, FAH, GBE1, ACSL1 | 0.00044 |
| TISSUES | BTO:0001488 | Endocrine gland | ALDH1A1, HINT1, TUBB6, PLEC, UGP2, PGM1, ASS1, FHL1, FAH, GBE1, ACSL1, PALLD, TXNRD1 | 0.00088 |
| TISSUES | BTO:0001491 | Viscus | ALDH1A1, HINT1, PLEC, UGP2, PGM1, ASS1, FHL1, FAH, GBE1, ACSL1, PALLD | 0.0124 |
| TISSUES | BTO:0003099 | Internal female genital organ | ALDH1A1, TUBB6, PLEC, UGP2, PGM1, ASS1, PALLD, TXNRD1 | 0.0289 |
| TISSUES | BTO:0000099 | Astrocyte | PGM1, TXNRD1 | 0.0415 |
| UniProt Keywords | KW-0007 | Acetylation | ALDH1A1, HINT1, PLEC, UGP2, PGM1, ASS1, FHL1, FAH, GBE1, ACSL1, TXNRD1 | 0.00054 |
| UniProt Keywords | KW-0322 | Glycogen storage disease | PGM1, GBE1 | 0.0439 |

**Supplementary Table 5**: STRING analysis of proteins which expression is significantly different between HLCs and NT-WJ-MSCs. *FDR= False Discovery Rate. This measure describes how significant the enrichment is. Shown are p-values corrected for multiple testing within each category using the Benjamini–Hochberg procedure.
