# Supplementary figures and images for: Characterization and Proteomic Profiling of Hepatocyte-like Cells Derived from Human Wharton’s Jelly Mesenchymal Stromal Cells: De Novo Expression of Liver-Specific Enzymes
Source: Biology (Basel). 2025 Jan 24;14(2):124. doi: 10.3390/biology14020124 (PMC11851833; doi:10.3390/biology14020124)

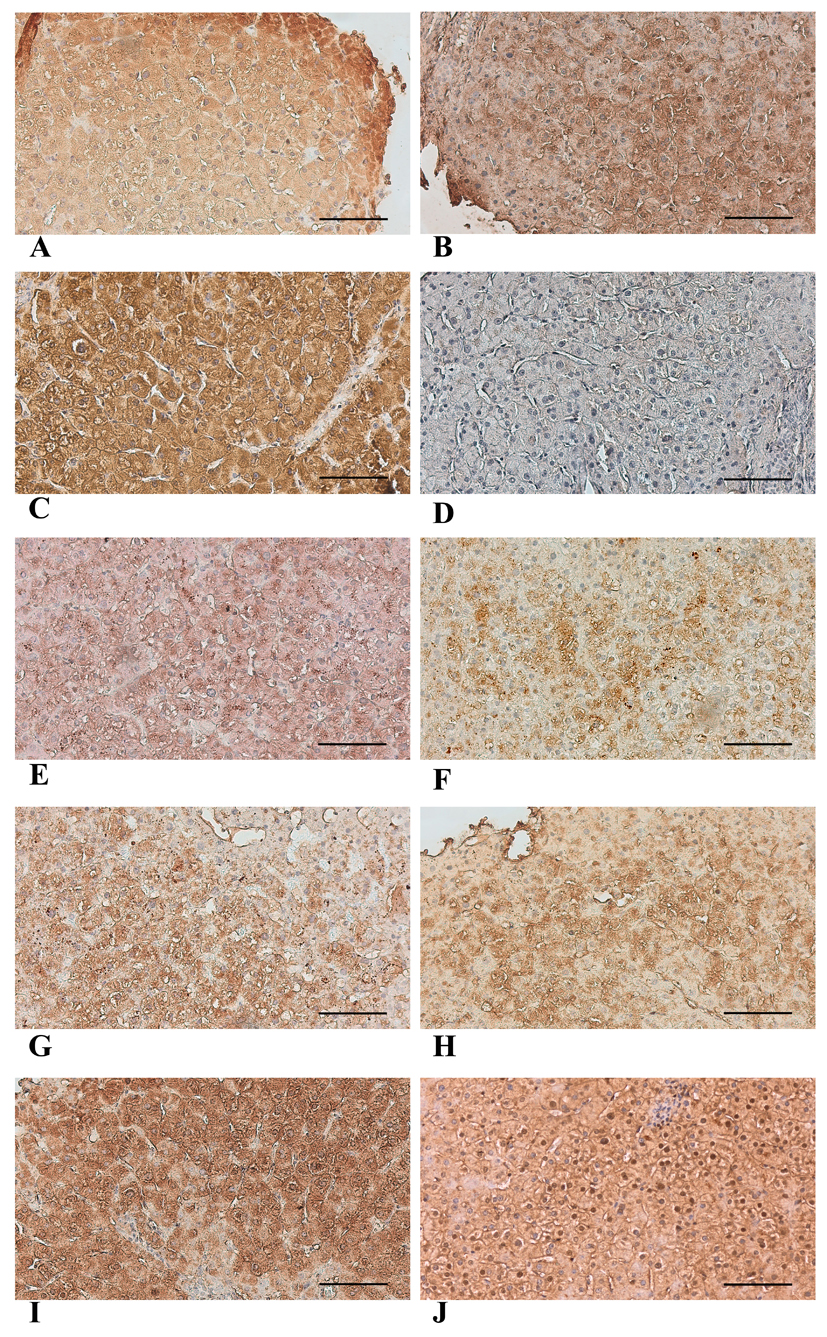

Supplement: Supplementary file 1 [file biology-14-00124-s001.zip › Figure S4.jpg]

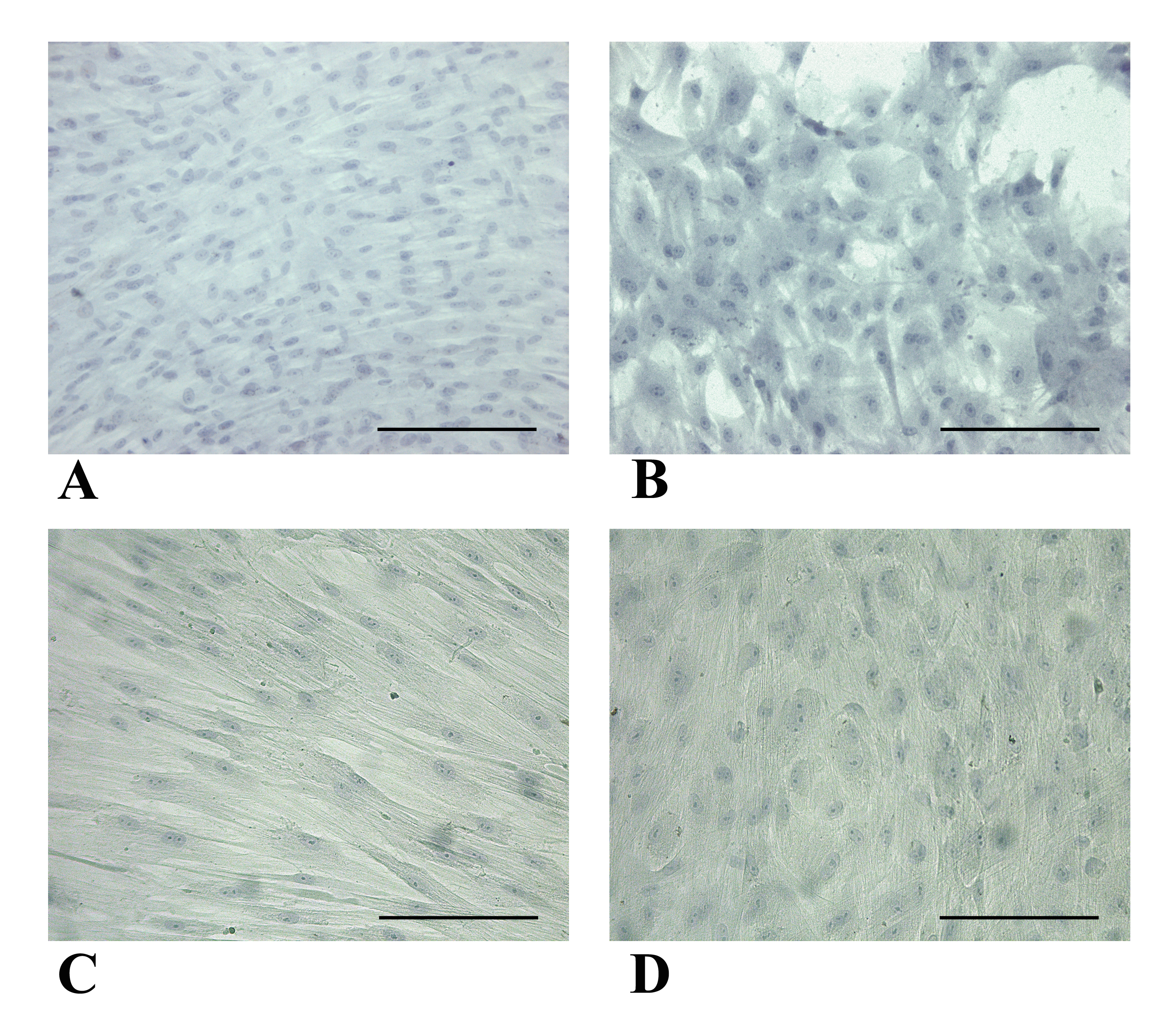

Supplement: Supplementary file 1 [file biology-14-00124-s001.zip › Figure S3.tif]

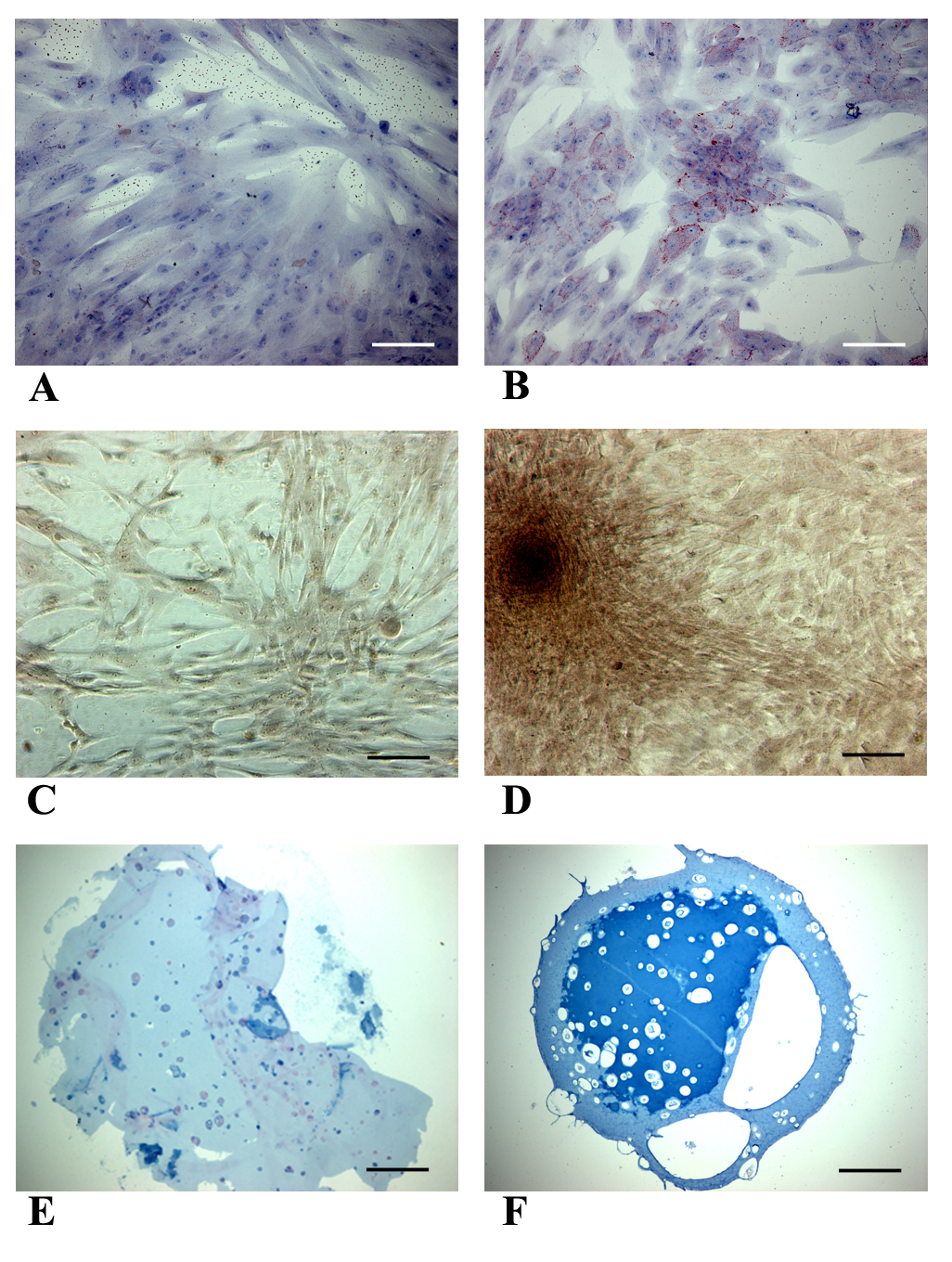

Supplement: Supplementary file 1 [file biology-14-00124-s001.zip › Figure S1.tif]

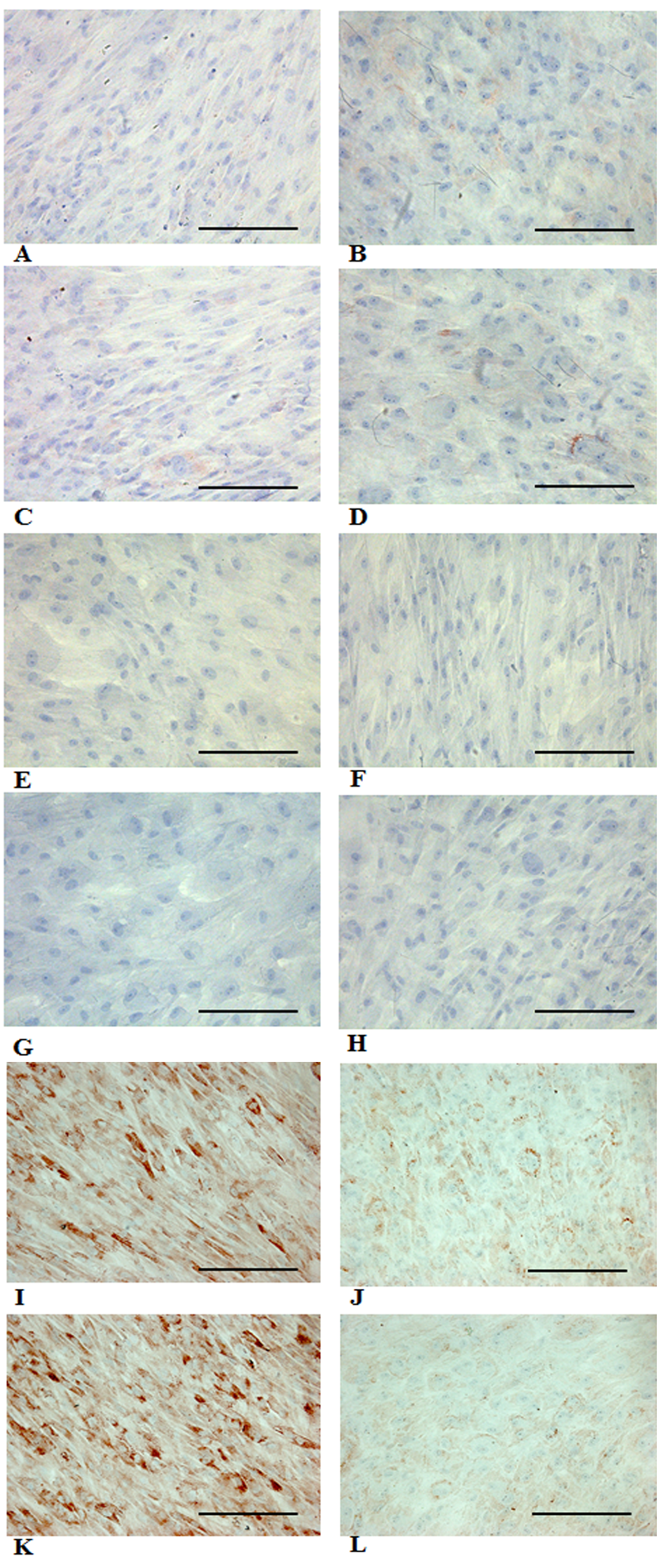

Supplement: Supplementary file 1 [file biology-14-00124-s001.zip › Figure S5.tif]

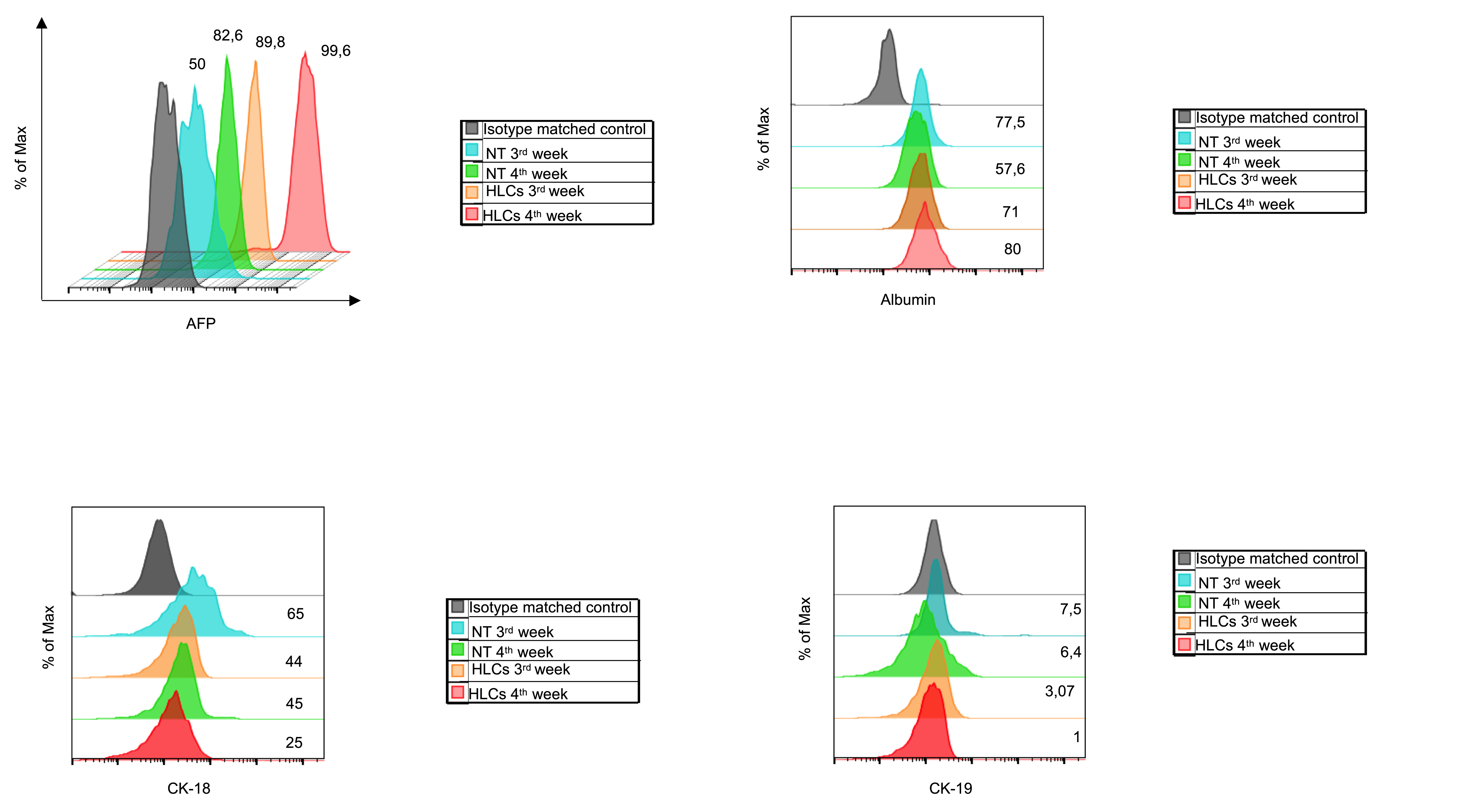

Supplement: Supplementary file 1 [file biology-14-00124-s001.zip › Figure S2.tiff]
